# Correction: Bees for Development: Brazilian Survey Reveals How to Optimize Stingless Beekeeping

**DOI:** 10.1371/journal.pone.0130111

**Published:** 2015-06-03

**Authors:** The PLOS ONE Staff

## Notice of Republication

This article was republished on May 15, 2015, to correct the legend for Table 1, which was mistakenly incorporated into the body text during the production process. The publisher apologizes for the error. Please download this article again to view the correct version. The originally published, uncorrected article and the republished, corrected article are provided here for reference.

## Supporting Information

S1 File Originally published, uncorrected article. (PDF)

S2 File Republished, corrected article. (PDF)
