# Supplementary figures and images for: Bees for Development: Brazilian Survey Reveals How to Optimize Stingless Beekeeping
Source: PLoS One. 2015 Mar 31;10(3):e0121157. doi: 10.1371/journal.pone.0121157 (PMC4380461; doi:10.1371/journal.pone.0121157)

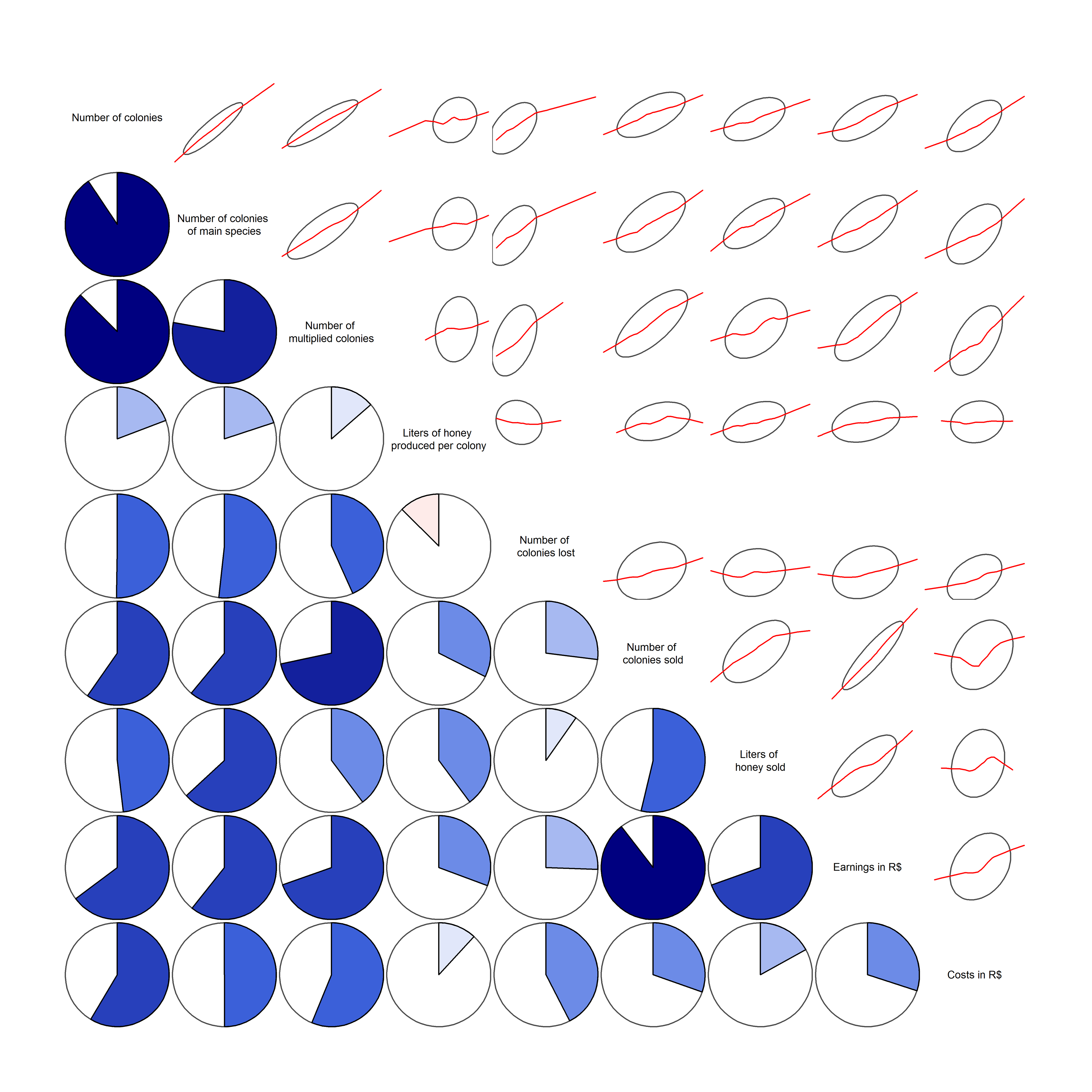

Supplement: S1 Fig — (TIF) [file pone.0121157.s002.tif]

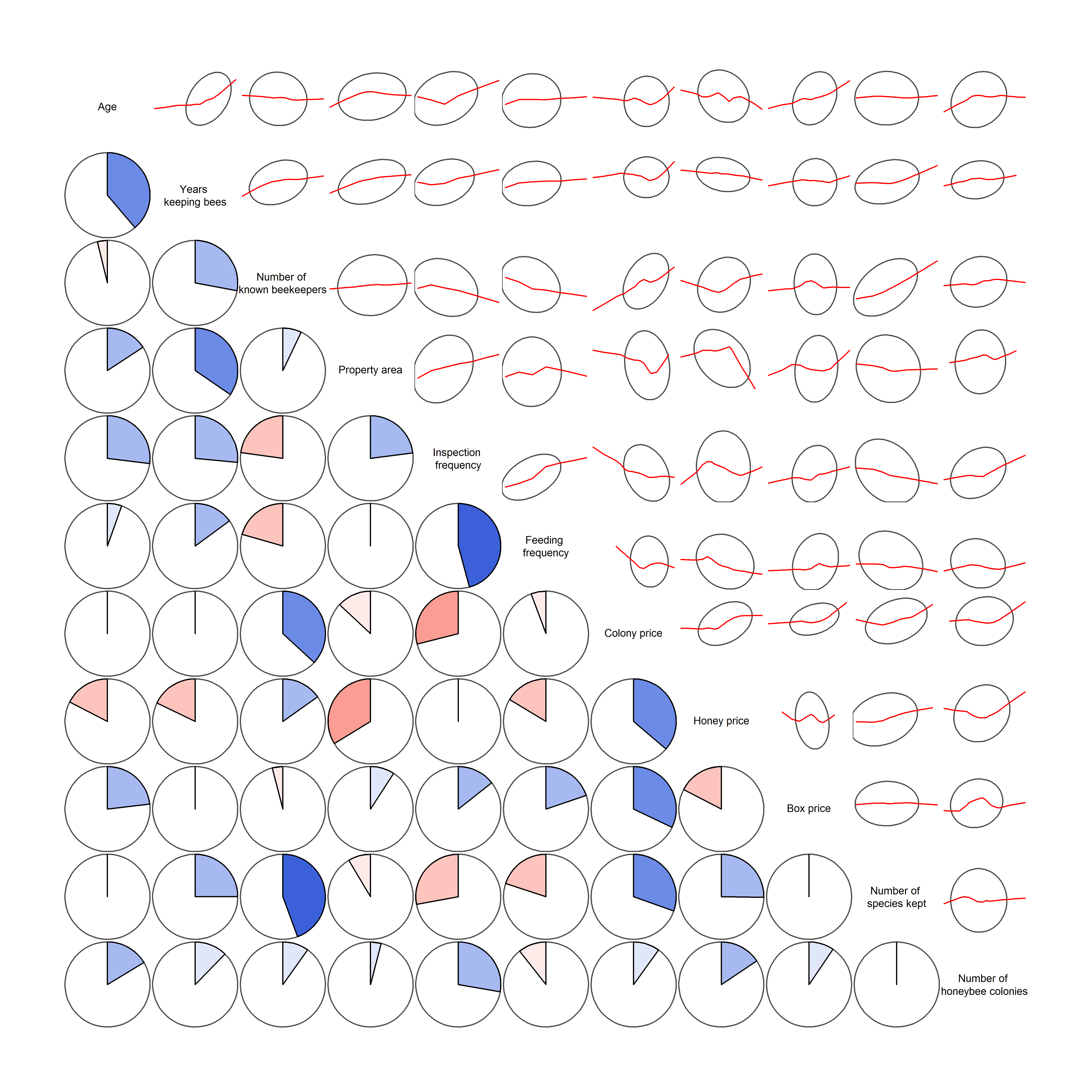

Supplement: S2 Fig — (TIF) [file pone.0121157.s003.tif]
