# Supplementary material for: Bees for Development: Brazilian Survey Reveals How to Optimize Stingless Beekeeping
Source: PLoS One. 2015 Mar 31;10(3):e0121157. doi: 10.1371/journal.pone.0121157 (PMC4380461; doi:10.1371/journal.pone.0121157)
Supplement: S1 Questionnaire — (PDF) [file pone.0121157.s004.pdf]

Brazilian stingless beekeeping diagnostic

Researcher / technician metadata

1. Name *

____

2. Professional affiliation *

____

3. Email *

____

4. Data collection date (date of interview) *

____

Beekeeper and property

5. Full name (and nickname) *

____

6. State *

____

7. County, city or place where the bees are kept *

____

8. Contact info (telephone / email)

____

9. Gender: ☐ M ☐ F

10. Age:

____

11. Education level

- ☐ no education
- ☐ literate
- ☐ elementary
- ☐ middle
- ☐ high
- ☐ graduation

12. Main job

____

13. Property type

- ☐ rural
- ☐ urban

14. Property size

____

15. Do you own the property?

☐ yes ☐ no

16. Where do you keep the bees?

- ☐ backyard
- ☐ rural property
- ☐ diferente places

17. Is there livestock in the property where the bees are kept (cattle, goats, poultry, fish, etc.)?

☐ yes ☐ no

18. Are there crops in the property where the bees are kept (corn, beans, manioc, sugarcane, etc.)?

☐ yes ☐ no

19. Are there flowering plants in the property where the bees are kept (fruit trees or other plants)?

☐ yes ☐ no

20. Is there native vegetation in the property where the bees are kept (or within 3 Km)?

 yes no

21. What is the main water source in the property where the bees are kept?

- River or lake
- pound or tank
- pipe water

22. Are pesticides used in the property where the bees are kept (insecticides or herbicides)?

 yes no

23. How long have you been keeping stingless bees?

____

24. Why do you keep stingless bees?

- to earn money selling honey or colonies
- to consume their honey
- to help preserve the bees
- as a hobby
- other:

25. Someone else keeps bees in the family?

 yes no

26. How did you learn to keep bees?

- alone
- with another beekeeper
- with a technitian / extension officer

27. Did you participate in a stingless beekeeping course?

 yes no

28. Do you know any website about stingless beekeeping?

 yes no

29. How many stingless beekeepers do you know?

____

30. In addition to keeping stingless bees, do you keep honeybees (*Apis mellifera*)?

 yes no

31. If you do keep honeybees (*Apis mellifera*), how many honeybee colonies do you have?

____

32. In your opinion, what is the main problem of keeping stingless bees?

- dry season/winter
- lack of technical skills
- deforestation
- lack of money to invest
- legislation
- other:

33. In your opinion, today there are more or less stingless bees in the wild than 50 years ago?

 today there are more bees

 today there are less bees

Stingless bee management

34. What is the main species you keep? *

Common name (scientific name)	Mark with an X
Jataí (*Tetragonisca angustula*)	
Mandaçaia (*Melipona quadrifasciata*)	
Tujuba (*Melipona rufiventris*)	
Uruçu Nordestina (*Melipona scutellaris*)	
Jandaíra (*Melipona subnitida*)	
Mirim, Mosquito, Jatí (*Plebeia* sp.)	
Manduri (*Melipona marginata*)	
Mandaguari, Canudo (*Scaptotrigona sp.*)	
Rajada (*Melipona asilvae*)	
Guaraipo (*Melipona bicolor*)	
Mandaçaia Nordestina (*Melipona mandacaia*)	
Cupira (*Partamona sp.*)	
Moça branca, Marmelada (*Frieseomellita sp.*)	
Uruçu Amarela (*Melipona flavolineata*)	
Uruçu Cinzenta (*Melipona fasciculata*)	
Uruçu boca de renda (*Melipona seminigra*)	
Other:	

35. How many colonies of this species do you have (only from the main species kept)?

____

36. How many colonies do you have overall, including all stingless bee species kept?

____

37. How many stingless be species do you keep?

____

38. What is the main type of box you employ?

- ☐ modular INPA / EMBRAPA
- ☐ modular Paulo Nogueira Neto
- ☐ horizontal long box (Nordestina)
- ☐ vertical long box
- ☐ tree branch or Calabash fruit
- ☐ other:

39. From what kind of wood are most of your boxes made?

____

40. Do you buy empty boxes?

☐ yes ☐ no

41. If you do buy empty boxes, how much do you pay for one?

R$_______________________________________

42. Do you check/inspect your bees?

☐ yes ☐ no

43. If you do check your bees, how frequently?

- ☐ dayly ☐ weekly
- ☐ biweekly ☐ monthly
- ☐ trimonthly ☐ half-yearly
- ☐ yearly

44. Do you feed your bees with sugar syrup or honey?

- yes
- no

45. If you do feed your bees, how frequently?

- dayly
- weekly
- biweekly
- monthly
- trimonthly
- half-yearly
- yearly

46. If you do feed your bees, where do you provide the food?

- inside the boxes
- outside the boxes

47. Have you lost any colony due to pests or predators?

- yes
- no

48. Do you use vinegar against parasitic flies (phorid flies)?

- yes
- no

49. Do you use grease/oil/poison against ants?

- yes
- no

50. Do you use any protection against lizards, frogs or birds (kills, has a cat, protection on nest entrances)?

- yes
- no

51. Did you lose any colony due to the insecticide sprayed by the dengue-fever car?

- yes
- no

52. How many colonies did you lose last year due to death or swarming?

____

53. How much money do you spend per year keeping stingless bees (buying boxes, new nests, food, etc.)?

R$_______________________________________

Multiplication

54. Do you multiply stingless bee nests? *

- yes
- no

55. How do you multiply nests?

- using a single brood disc
- using two or more brood discs from the same nest
- using two or more brood discs from different nests

56. Do you feed newly multiplied (daughter) colonies?

- yes
- no

57. How many new (daughter) colonies did you produce last year?

____

58. Which colonies do you select for multiplying?

- only the strongest/more productive
- any colony, even the weak ones

Honey sales

59. Do you sell stingless bee honey? *

- yes
- no

60. How do you harvest honey?

- piercing honey-pots and "fipping the box"
- using a syringe
- using an electric pump connected to a plastic hose

61. Do you use any honey conservation method?

- I do not use any method
- pasteuriztion
- maturation
- dehumidification / dehydration
- freezing
- I leave honey in the regrigerator

62. On average, how many liters of honey are produced by a single colony in a year? (from the main species kept)

____

63. How many liters of honey did you sell last year?

____

64. How much do you charge for one liter of honey of your main species kept?

R$_______________________________________

65. Who normally buys the honey?

- ☐ particular client
- ☐ merchant or store

66. How do you sale the honey?

- ☐ in labeled containers
- ☐ in unlabeled containers

67. Where do you sale the honey?

- ☐ at home
- ☐ in a road post or store
- ☐ in a supermarket

68. Do you participate in a cooperative/local organization selling honey?

☐ yes ☐ no

Colony sales

69. Do you sell stigless bee colonies? *

☐ yes ☐ no

70. How do you normally sale the colonies?

- ☐ in boxes
- ☐ in tree branches or Calabash fruits

71. How much do you charge for one colony of the main species kept?

R$_______________________________________

72. How many colonies did you sale last year?

____

73. Who normally buys the colonies?

- ☐ hobbyists/amateurs
- ☐ other stingless beekeepers
- ☐ research/develpment institutes

Additional observations

74. Do you sell any other stingless bee product?

- ☐ I do not sell any other product
- ☐ polen
- ☐ propolis (geopropolis)
- ☐ wax
- ☐ other:

75. Additional observations (any relevant comments)

____

____

____

____

Informed Participation Consent

Herewith I agree to participate in the Project “Brazilian stingless beekeeping diagnostic”, and I understand that private information such as the names and addresses of participants will not be disclosed to protect their privacy.

____

(signature of the interviewed beekeeper)

Please send to:
Dr. Rodolfo Jaffé
Laboratório de Abelhas, Instituto de Biociências,
Universidade de São Paulo (USP).
Rua do Matão 321, 05508-090 São Paulo-SP.

Questions and suggestions:
Dr. Rodolfo Jaffé
Tel: (11) 9 4962 0915
Email: r.jaffe@ib.usp.br
