# Supplementary material for: Bees for Development: Brazilian Survey Reveals How to Optimize Stingless Beekeeping
Source: PLoS One. 2015 Mar 31;10(3):e0121157. doi: 10.1371/journal.pone.0121157 (PMC4380461; doi:10.1371/journal.pone.0121157)
Supplement: S1 Table — (PDF) [file pone.0121157.s005.pdf]

S1 Table: Description of all response and predictor variables.

	Variable	Description
**Response**	Multiplies colonies?	Whether beekeepers ever multiply their colonies
	Sells colonies?	Whether beekeepers ever sale their colonies
	Sells honey?	Whether beekeepers ever sale honey
	Number of colonies	Total number of colonies kept
	Number of colonies of main species	Number of colonies of the main species kept
	Number of multiplied colonies	Number multiplied colonies in a year, among beekeepers that multiplied at least one colony during the previous year.
	Liters of honey produced per colony	Mean volume of honey produced by a colony of the main kept species per year (in liters).
	Number of colonies lost	Number of colonies lost in a year
	Number of colonies sold	Number of colonies sold in a year, among beekeepers that sold at least one colony during the previous year.
	Liters of honey sold	Volume of honey sold in a year, among beekeepers that sold honey during the previous year.
	Earnings is R$	Yearly income from selling honey and/or colonies, calculated as: Earnings = (Liters of honey sold x Price of 1lt of honey) + (Number of colonies sold x Price of one colony)
	Costs in R$	Yearly costs associated to stingless beekeeping
**Predictor**	Age	Age of the beekeepers
	Years keeping bees	Years keeping stingless bees
	Number of known beekeepers	Number of beekeepers known personally or through social networks
	Meliponiculture course	If beekeepers participated in a meliponiculture course
	Meliponiculture website	If beekeepers know a website related to meliponiculture
	Education level	Level of education attained: None/Elementary, Middle, High or Graduate
	Initiation in meliponiculture	If beekeepers were first initiated in the practice alone, with the help of a fellow beekeeper, or through an extension officer
	Property area	The area of the property were the bees are kept
	Property type	If the property was rural or urban
	Cattle	Presence/absence of cattle in the property
	Crops	Presence/absence of crops in the property
	Native vegetation	Presence/absence of native vegetation within a 3Km ratio.
	Pesticides	Use/disuse of pesticides in the property
	Water source	Pipe water, pound/tank, or river
	Inspection frequency	How frequent beekeepers inspect their colonies: Daily, weekly, quarterly, monthly, trimonthly, half-yearly, or yearly
	Selective breeding	Weather beekeepers avoid mixing brood combs from different nests when multiplying colonies, and thus are able to maintain maternal lines
	Main box type employed	Horizontal long box (north-eastern model), modular box, or other types of boxes
	Honey harvest method	Weather beekeepers collect honey by tearing the honey pots and “flipping the box” to let the honey drain into a collection container, or by using syringes or electric pumps connected to plastic hoses
	Feeding frequency	How frequent beekeepers feed their colonies: Daily, weekly, quarterly, monthly, trimonthly, half-yearly, or yearly
	Supplementary	Whether beekeepers feed their colonies with sugar syrup/honey or not

	feeding	
	Feeding place	If sugar syrup is placed inside or outside colonies
	Feeding of multiplied colonies	If newly multiplies colonies are provided with sugar syrup or not
	Use of vinegar	If vinegar traps are used or not to control parasitic flies
	Use of oil or grease	If grease or oil is used or not to protect colonies from ants and other insects
	Other pest control	If pests such as lizards, frogs or birds are controlled in any way or not
	Colony price	The average price of a living colony
	Honey price	The average price of one liter of honey of the main species kept
	Box price	The average price of an empty box
	Property ownership	If the beekeeper is the owner of the property where the bees are kept
	Beekeepers in the family	If there are other family members that keep bees too
	Place were the bees are kept	If the bees are kept at the home´s backyard, in a rural property, or I several places
	Purchase of boxes	If beekeepers buy empty boxes or not
	Use of labels in honey containers	If beekeepers label the honey containers they intend to sell
	Participates in a honey cooperative	If beekeepers participate in a honey cooperative or not
	Customers buying colonies	If the customers buying colonies are hobbyists or established beekeepers
	Honey conservation method	Which method is employed to preserve honey: None, placing honey in a refrigerator, or using one of the established honey conservation methods (freezing, pasteurization, maturation or dehumidification)
	Number of species	Number of species kept
	Number of honeybee colonies	Number of honeybee colonies (*Apis mellifera*) kept, among stingless beekeepers that also keep honeybees
	Keeping of honeybees	If beekeepers keep *Apis mellifera* as well as stingless bees
