# Supplementary material for: Bees for Development: Brazilian Survey Reveals How to Optimize Stingless Beekeeping
Source: PLoS One. 2015 Mar 31;10(3):e0121157. doi: 10.1371/journal.pone.0121157 (PMC4380461; doi:10.1371/journal.pone.0121157)
Supplement: S2 Table — (PDF) [file pone.0121157.s006.pdf]

**S2 Table:** Gender, State, and Job distribution of interviewed beekeepers.

| Item | Category | N |
|---|---|---|
| **Gender** | M | 237 |
| | F | 14 |
| **State** | SP | 36 |
| | PR | 26 |
| | SC | 26 |
| | BA | 23 |
| | RN | 22 |
| | MG | 19 |
| | PA | 18 |
| | RS | 17 |
| | PE | 16 |
| | CE | 11 |
| | RJ | 10 |
| | PB | 8 |
| | DF | 4 |
| | ES | 4 |
| | AL | 2 |
| | AM | 2 |
| | GO | 2 |
| | MA | 2 |
| | MS | 2 |
| | RO | 1 |
| **Job**[a] | Agronomy | 64 |
| | Other | 48 |
| | Public Server | 29 |
| | Retired | 21 |
| | Commerce | 17 |
| | Tradesman | 14 |
| | Education | 10 |
| | Health | 9 |
| | Student | 8 |
| | Technitian | 7 |
| | Administration | 6 |

[a] Not all beekeepers disclosed their main economic activity.
