# Supplementary material for: Bees for Development: Brazilian Survey Reveals How to Optimize Stingless Beekeeping
Source: PLoS One. 2015 Mar 31;10(3):e0121157. doi: 10.1371/journal.pone.0121157 (PMC4380461; doi:10.1371/journal.pone.0121157)
Supplement: S3 Table — Ntot represents the total number of beekeepers providing an answer for each question (not all beekeepers responded to all questions), while Nreply represents the number of beekeepers in each reply category (only for categorical variables). (PDF) [file pone.0121157.s007.pdf]

S3 Table: Summary statistics for all variables. N_{tot} represents the total number of beekeepers providing an answer for each question (not all beekeepers responded to all questions), while N_{reply} represents the number of beekeepers in each reply category (only for categorical variables).

	Variable	N_{tot}	N_{reply}	Median/Mean	Min-Max
**Response**	Multiplies colonies?	251	No: 48<br>Yes: 203	-	-
	Sells colonies?	251	No: 177<br>Yes: 74	-	-
	Sells honey?	251	No: 156<br>Yes: 95	-	-
	Number of colonies	251	-	22.00/72.65	1-3500
	Number of colonies of main species	241	-	13.00/36.66	1-800
	Number of multiplied colonies	165	-	8.00/19.85	1-200
	Honey production per colony	89	-	1.50/2.07	0.04-10.00
	Colony loses	234	-	2.00/7.265	0-700
	Colony sales	63	-	12.00/24.33	1-200
	Honey sales	76	-	14.50/40.03	1-500
	Income	102	-	1500/4535	70-55000
	Costs	168	-	500/1511	15-23600
**Predictor**	Age	247	-	46.00/44.60	15-80
	Years keeping bees	236	-	6/10.86	0.083-54
	Number of known beekeepers	217	-	8/22.87	1-200
	Meliponiculture course	224	No: 102<br>Yes: 122	-	-
	Meliponiculture website	248	No: 63<br>Yes: 185	-	-
	Education level	249	None/Elementary: 58<br>Middle: 74<br>High: 73<br>Graduate: 44	-	-
	Initiation in meliponiculture	246	Beekeeper: 96<br>Alone: 109<br>Technitian: 41	-	-
	Property area	169	-	2.00/19.90	0.004-300
	Property type	234	Rural: 124<br>Urban: 110	-	-
	Cattle	249	No: 153<br>Yes: 96	-	-
	Crops	249	No: 135<br>Yes: 114	-	-
	Native vegetation	241	No: 32<br>Yes: 209	-	-
	Pesticides	245	No: 208<br>Yes: 37	-	-
	Water source	243	Pipe water: 81<br>Pound/tank: 48<br>River: 114	-	-
	Inspection frequency	239	Dayly: 31<br>Weekly: 79<br>Quarterly: 51<br>Monthly: 45<br>Trimonthly: 9<br>Half-yearly: 11<br>Yearly: 13	-	-

	Selective breeding	196	No: 50<br>Yes: 146	-	-
	Main box type employed	232	Horizontal long box: 49<br>Modular box: 128<br>Others: 55	-	-
	Honey harvest method	94	Flipping the box: 42<br>Syringe/Pump: 52	-	-
	Feeding frequency	174	Dayly: 10<br>Weekly: 88<br>Quarterly: 29<br>Monthly: 27<br>Trimonthly: 7<br>Half-yearly: 4<br>Yearly: 9	-	-
	Supplementary feeding	244	No: 72<br>Yes: 172	-	-
	Feeding place	163	Inside: 140<br>Outside: 23	-	-
	Feeding of multiplied colonies	199	No: 21<br>Yes: 178	-	-
	Use of vinegar	248	No: 116<br>Yes:132	-	-
	Use of oil or grease	249	No: 158<br>Yes: 91	-	-
	Other pest control	244	No: 152<br>Yes: 92	-	-
	Colony price	73	-	175/193.4	20-500
	Honey price	90	-	50/67.22	15-200
	Box price	122	-	40/43.02	10-110
	Property ownership	231	No: 53<br>Yes: 178	-	-
	Beekeepers in the family	249	No: 165<br>Yes: 84	-	-
	Place were the bees are kept	247	At home: 120<br>Rural property: 61<br>Several places: 66	-	-
	Purchase of boxes	245	No: 116<br>Yes: 129	-	-
	Use of labels in honey containers	95	No: 63<br>Yes: 32	-	-
	Participates in a honey cooperative	95	No: 71<br>Yes: 24	-	-
	Customers buying colonies	69	Hobbyists: 34<br>Beekeepers: 35	-	-
	Honey conservation method	93	None: 34<br>Refrigerator: 31<br>Established: 28	-	-
	Number of species	243	-	4/5	1-28
	Number of honeybee colonies	79	-	20/54.29	2-600
	Keeping of honeybees	233	No: 149<br>Yes: 84	-	-
