# Supplementary material for: Bees for Development: Brazilian Survey Reveals How to Optimize Stingless Beekeeping
Source: PLoS One. 2015 Mar 31;10(3):e0121157. doi: 10.1371/journal.pone.0121157 (PMC4380461; doi:10.1371/journal.pone.0121157)
Supplement: S4 Table — Although beekeepers also reared other species, they were not included in this list since they were not registered as the main species kept. (PDF) [file pone.0121157.s008.pdf]

S4 Table: List of the main species kept by 246 Brazilian stingless beekeepers, their common names in Brazil, and the number and proportion of beekeepers rearing each one. Although beekeepers also reared other species, they were not included in this list since they were not registered as the main species kept.

Species [a]	Popular name(s) in Brazil [a]	N. beekeepers (%)
Tetragonisca angustula Latreille, 1811	jataí	69 (28,0%)
Melipona quadrifasciata Lepeletier, 1836	mandaçaia	43 (17,5%)
Melipona subnitida Ducke, 1909	jandaíra	33 (13,4%)
Melipona scutellaris Latreille, 1811	uruçu do nordeste	28 (11,4%)
Melipona fasciculata Smith, 1854	uruçu-cinzenta, tiúba	14 (5,7%)
Melipona mandacaia Smith 1863	mandaçaia	11 (4,5%)
Melipona flavolineata Friese, 1900	uruçu-amarela; ira-açu	11 (4,5%)
Melipona aff. *rufiventris* Lepeletier, 1836	tujuba; tuiúva; tujuva	10 (4,1%)
Plebeia spp. Schwarz, 1938	mirim, mosquito, jati, mosquitinho	8 (3,3%)
Scaptotrigona spp. Moure, 1942	canudo, mandaguari, tubiba, tubi, tubuna	6 (2,4%)
Melipona bicolor Lepeletier, 1836	guaraipo	3 (1,2%)
Melipona seminigra Friese, 1903	uruçu-boca-de-renda	2 (0,8%)
Frieseomelitta spp. Ihering, 1912	moça branca, moça preta, amarela, marmelada, breu	2 (0,8%)
Nannotrigona testaceicornis (Lepeletier, 1836)	iraí	1 (0,4%)
Melipona marginata Lepeletier, 1836	manduri	1 (0,4%)
Melipona interrupta Latreille, 1811	jandaíra-preta-da-Amazônia; jupará	1 (0,4%)
Melipona capixaba Moure & Camargo, 1994	uruçu-preto	1 (0,4%)
Melipona favosa Fabricius, 1798	rabipintada; erica	1 (0,4%)
Friesella spp. Moure, 1946	mirim preguiça	1 (0,4%)

[a] References:

Camargo MF, Pedro SRM (2012) Meliponini Lepeletier, 1836. Catalogue of Bees (Hymenoptera, Apoidea) in the Neotropical Region. Online version available at http://www.moure.cria.org.br/catalogue

Nogueira-Neto P (1997) Vida e Criação de Abelhas Indígenas Sem Ferrão. Editora Nogueirapis, São Paulo. Online version available at http://www.webbee.org.br/webbee123/publicacoes/livro_pnn.pdf
