# Supplementary material for: Bees for Development: Brazilian Survey Reveals How to Optimize Stingless Beekeeping
Source: PLoS One. 2015 Mar 31;10(3):e0121157. doi: 10.1371/journal.pone.0121157 (PMC4380461; doi:10.1371/journal.pone.0121157)
Supplement: S5 Table — Estimates are provided for each level of the categorical predictors. Note that all predictors included in these models significantly increased the model´s likelihood at p < 0.05 (likelihood ratio tests). (PDF) [file pone.0121157.s009.pdf]

S5 Table: Regression estimates, standard errors (SE), confidence intervals (CI), *z* or *t*-values and *p*-values for all best models. Estimates are provided for each level of the categorical predictors. Note that all predictors included in these best models significantly increased the model´s likelihood at $p < 0.05$ (likelihood ratio tests).

Response	Predictor	Estimate (SE)	CI	*z/t*	*p* [a]	r^2 [b]
**Multiplies colonies?**	Number of colonies (log)	0.44 (0.19)	0.09, 0.82	2.39	0.017 / 0.02	0.43
	Meliponiculture course (Yes)	1.25 (0.49)	0.31, 2.27	2.54	0.011 / 0.02	
	Native vegetation (Yes)	1.57 (0.54)	0.52, 2.66	2.89	0.004 / 0.008	
	Supplementary feeding (Yes)	1.64 (0.44)	0.79, 2.54	3.71	<0.001 / <0.001	
**Sells colonies?**	Honey sales (Yes)	1.73 (0.42)	0.96, 2.60	4.16	<0.001	0.35
	Number of known beekeepers (log)	0.32 (0.16)	0.01, 0.62	2.03	0.04 / 0.04	
	Supplementary feeding (Yes)	1.12 (0.45)	0.27, 2.06	2.47	0.01 / 0.03	
**Sells honey?**	Colony sales (Yes)	1.65 (0.47)	0.75, 2.63	3.48	<0.001	0.57
	Years keeping bees (log)	0.47 (0.21)	0.07, 0.90	2.23	0.03 / 0.05	
	Meliponiculture course (Yes)	0.97 (0.44)	0.12, 1.85	2.21	0.03 / 0.03	
	Education (Middle)	-0.08 (0.62)	-1.28, 1.16	-0.14	0.89 / 1.0	
	Education (High)	-2.08 (0.68)	-3.49, 0.78	-3.05	0.002 / 0.01	
	Education (Graduate)	-1.51 (0.69)	-2.90, -0.16	-2.17	0.03 / 0.14	
	Crops (Yes)	1.16 (0.44)	0.31, 2.06	2.62	0.01	
	Property type (Urban)	-0.98 (0.45)	-1.90, -0.11	-2.18	0.03	
**Number of colonies**	Years keeping bees (log)	0.62 (0.06)	0.51, 0.74	10.53	<0.001 / <0.001 (141.13)	0.62
	Number of known beekeepers (log)	0.33 (0.06)	0.22, 0.44	5.78	<0.001 /<0.001 (173.11)	
	Native vegetation (Yes)	0.51 (0.18)	0.15, 0.87	2.76	0.006 / 0.008 (180.94)	
	Use of vinegar (Yes)	0.46 (0.13)	0.2, 0.72	3.46	<0.001 (180.91)	
	Supplementary feeding (Yes)	0.62 (0.16)	0.31, 0.92	3.91	<0.001 / <0.001 (178.48)	
**Number of colonies of main species**	Years keeping bees (log)	0.54 (0.06)	0.41, 0.67	8.38	<0.001 / <0.001 (190.45)	0.45
	Number of known beekeepers (log)	0.31 (0.06)	0.2, 0.43	5.30	<0.001 / <0.001 (190.97)	
**Number of multiplied colonies**	Number of colonies (log)	0.85 (0.04)	0.77, 0.94	19.44	<0.001 / <0.001 (117.72)	0.84
	Number of known beekeepers (log)	0.12 (0.04)	0.04, 0.2	2.85	0.01 / 0.01 (116.50)	
	Supplementary feeding (Yes)	0.34 (0.13)	0.08, 0.6	2.53	0.01 / 0.03 (120.92)	
	Property ownership (Yes)	-0.34 (0.13)	-0.58, -0.09	-2.67	0.01 (118.50)	
**Liters of honey produced per colony**	Selective breeding (Yes)	0.38 (0.18)	0.02, 0.74	2.11	0.04 (67.13)	0.24
**Number of colonies lost**	Inspection frequency	0.24 (0.09)	0.07, 0.42	2.69	0.01	0.1
	Honey harvest method (Syringe/Pump)	-0.61 (0.31)	-1.24, 0.002	-1.95	0.05	
**Number of colonies sold**	Years keeping bees (log)	0.82 (0.12)	0.59, 1.06	6.99	<0.001 / <0.001 (55.56)	0.51

**Liters of honey sold**	Number of colonies of main species (log)	0.45 (0.11)	0.23, 0.68	3.92	<0.001 (53.55)	0.74
	Years keeping bees (log)	0.61 (0.27)	0.08, 1.2	2.25	0.06 / 0.06 (7.53)	
	Honey conservation method (Refrigerator)	-0.32 (0.26)	-0.86, 0.19	-1.22	0.23 / 0.46 (43.60)	
	Honey conservation method (Established)	0.5 (0.26)	-0.04, 1	1.92	0.06 / 0.18 (52.84)	
**Earnings is R$**	Number of colonies (log)	0.41 (0.14)	0.14, 0.68	2.99	0.003 / 0.008	0.63
	Labeling of honey containers (Yes)	-1.00 (0.91)	-2.82, 0.82	-1.10	0.28	
	Honey conservation method (Refrigerator)	-0.75 (0.3)	-1.34, -0.16	-2.54	0.01 / 0.05	
	Honey conservation method (Established)	0.31 (0.31)	-0.31, 0.93	1.01	0.32 / 0.46	
	Number of colonies (log): Labeling of honey containers (Yes)	0.47 (0.21)	0.06, 0.88	2.30	0.02	
**Costs in R$**	Number of colonies (log)	0.47 (0.06)	0.35, 0.6	7.33	<0.001 / <0.001	0.46
	Education (Middle)	-0.02 (0.31)	-0.62, 0.59	-0.05	0.96 / 1.0	
	Education (High)	0.51 (0.3)	-0.08, 1.11	1.70	0.09 / 0.27	
	Education (Graduate)	0.75 (0.34)	0.08, 1.41	2.23	0.03 / 0.14	
	Feeding frequency (centered)	-0.17 (0.06)	-0.3, -0.04	-2.67	0.01	
	Age (centered)	0.02 (0.01)	0.01, 0.03	2.84	0.01	
	Feeding frequency (centered) : Age (centered)	0.01 (0.01)	0.003, 0.02	2.62	0.01	

[a] For predictors appearing more than once in the best models, we computed *p-values* adjusted for multiple tests (Holm-corrected *p-values* are given after /). Degrees of freedom are given in brackets for cases were *p-values* were computed using the Satterthwaite approximation (lmerTest package). [b] While conditional r^2 values are presented for Generalized Mixed-Effect Models (LMM and GLMM), adjusted r^2 are shown for linear models (LM).
