# Supplementary material for: Correction: Bees for Development: Brazilian Survey Reveals How to Optimize Stingless Beekeeping
Source: PLoS One. 2015 Jun 3;10(6):e0130111. doi: 10.1371/journal.pone.0130111 (PMC4454511; doi:10.1371/journal.pone.0130111)
Supplement: S2 File — (PDF) [file pone.0130111.s002.pdf]

PLOS ONE

RESEARCH ARTICLE

Bees for Development: Brazilian Survey Reveals How to Optimize Stingless Beekeeping

Rodolfo Jaffé[1]*, Nathaniel Pope[2], Airton Torres Carvalho[3], Ulysses Madureira Maia[3], Betina Blochtein[4], Carlos Alfredo Lopes de Carvalho[5], Gislene Almeida Carvalho-Zilse[6], Breno Magalhães Freitas[7], Cristiano Menezes[8], Márcia de Fátima Ribeiro[9], Giorgio Cristino Venturieri[8], Vera Lucia Imperatriz-Fonseca[1,3]

1 Departamento de Ecologia, Universidade de São Paulo. Rua do Matão 321, São Paulo-SP, Brazil, **2** Department of Integrative Biology, 401 Biological Laboratories, University of Texas, Austin, Texas, United States of America, **3** Departamento de Ciências Animais, Universidade Federal Rural do Semi-Árido, Avenida Francisco Mota 572, Mossoró-RN, Brazil, **4** Laboratório de Entomologia, Pontifícia Universidade Católica do Rio Grande do Sul, Av. Ipiranga, 6681, Caixa Postal 1429, Porto Alegre-RS, Brazil, **5** Insecta Research Group, Centro de Ciências Agrárias, Ambientais e Biológicas, UFRB, C. Postal: 118, Cruz das Almas-BA. Brazil, **6** Grupo de Pesquisas em Abelhas, Instituto Nacional de Pesquisas da Amazônia, Avenida André Araújo 2936, Caixa Postal 478, Manaus-AM, Brazil, **7** Departamento de Zootecnia—CCA, Universidade Federal do Ceará. Campus Universitário do Pici, Bloco 808, Fortaleza-CE, Brazil, **8** Embrapa Amazônia Oriental, Empresa Brasileira de Pesquisa Agropecuária, Belém-PA, Brazil, **9** Embrapa Semiárido, Empresa Brasileira de Pesquisa Agropecuária, Petrolina-PE, Brazil

* r.jaffe@ib.usp.br

CrossMark
click for updates

OPEN ACCESS

Citation: Jaffé R, Pope N, Carvalho AT, Maia UM, Blochtein B, de Carvalho CAL, et al. (2015) Bees for Development: Brazilian Survey Reveals How to Optimize Stingless Beekeeping. PLoS ONE 10(3): e0121157. doi:10.1371/journal.pone.0121157

Academic Editor: Wolfgang Blenau, University of Cologne, GERMANY

Received: October 1, 2014

Accepted: January 28, 2015

Published: March 31, 2015

Copyright: © 2015 Jaffé et al. This is an open access article distributed under the terms of the Creative Commons Attribution License, which permits unrestricted use, distribution, and reproduction in any medium, provided the original author and source are credited.

Data Availability Statement: All relevant data are within the paper and its Supporting Information files.

Funding: Fundação de Amparo à Pesquisa do Estado de São Paulo—FAPESP (RJ, 2012/13200-5), funded the study design, some data collection, data analysis, and the preparation and publication of this manuscript. Empresa Brasileira de Pesquisa Agropecuária—EMBRAPA (02.11.01.029.00.00), funded some data collection. Conselho Nacional de Desenvolvimento Científico e Tecnológico—CNPq, supported ATC and BMF, while the National Science Foundation—NSF, supported NP.

Abstract

Stingless bees are an important asset to assure plant biodiversity in many natural ecosystems, and fulfill the growing agricultural demand for pollination. However, across developing countries stingless beekeeping remains an essentially informal activity, technical knowledge is scarce, and management practices lack standardization. Here we profited from the large diversity of stingless beekeepers found in Brazil to assess the impact of particular management practices on productivity and economic revenues from the commercialization of stingless bee products. Our study represents the first large-scale effort aiming at optimizing stingless beekeeping for honey/colony production based on quantitative data. Survey data from 251 beekeepers scattered across 20 Brazilian States revealed the influence of specific management practices and other confounding factors over productivity and income indicators. Specifically, our results highlight the importance of teaching beekeepers how to inspect and feed their colonies, how to multiply them and keep track of genetic lineages, how to harvest and preserve the honey, how to use vinegar traps to control infestation by parasitic flies, and how to add value by labeling honey containers. Furthermore, beekeeping experience and the network of known beekeepers were found to be key factors influencing productivity and income. Our work provides clear guidelines to optimize stingless beekeeping and help transform the activity into a powerful tool for sustainable development.

Competing Interests: The authors have declared that no competing interests exist.

Introduction

Beekeeping should be regarded as a prime tool to achieve sustainable development [1–5]. Keeping bees can help low-income communities earn additional revenues from selling bee products, thus reducing the need to exploit other natural resources and creating incentives to protect natural habitats as food sources and nesting sites for the bees. Moreover, beekeeping contributes to the provision of pollination services, assuring crop yields and helping maintain plant biodiversity in natural ecosystems [6–9].

Although the commercial use of the honeybee (named Apiculture, referring to bees of the *Apis* genus) has become a major global business, the profitable use of stingless bees (designated Meliponiculture, after bees from the Meliponini tribe) has received much less attention [1, 10, 11]. Different indigenous tribes have exploited stingless bee products since ancient times, including the Maya from Mexico and Guatemala, the Kayapó from the Brazilian Amazon basin, the Abayandas pygmy from Uganda, and several Australian Aboriginal tribes [1, 12]. Currently, many stingless bee species are managed in the Americas, Africa, Asia and Australia, but across developing countries meliponiculture remains essentially informal, technical knowledge is scarce, and management practices lack standardization [1, 3, 13–15]. Commercialized bee products, including honey, colonies, and in a few cases crop pollination services, are generally unregulated, and demand often exceeds supply [3, 16, 17]. Meliponiculture thus remains a largely under-exploited business.

Stingless bees are an important asset to fulfill the growing agricultural demand for pollination, because they could compensate for the worldwide declines in honeybee populations [18–20] by assuring enough pollinators [21] and by pollinating crops more effectively [22]. In addition, remarkable features make stingless bees appealing both to potential entrepreneurs and development officers. Firstly, they are part of the local biodiversity of many tropical and subtropical ecosystems [23–25] and hence well adapted to local conditions. Secondly, stingless bees are key pollinators of both natural flora and commercial crops, and therefore of great biological and economic importance [6, 9, 26, 27]. Thirdly, lacking a functional stinger they cannot sting, thus making management easier (most stingless beekeepers never employ veils) and facilitating their use in confined conditions such as greenhouses [6]. Finally, technical skills and not physical strength determine the ability of beekeepers to manage stingless bees, which makes meliponiculture accessible to persons that would not be able to run an apiary by themselves.

Making meliponiculture a more profitable activity could attract new entrepreneurs and thus increase its relevance as a mean to achieve sustainable development. This, however, requires the optimization of management practices in order to increase the production of bee products and raise income from their sells. Achieving such optimization is hard, given the huge diversity of management practices (tightly linked to the cultural heritage), as well as the striking biological differences among species [12, 28]. Moreover, the different organizations that offer training in meliponiculture, usually rely on the personal experience of successful beekeepers rather than on quantitative data. Important efforts have been directed to train beekeepers and standardize management practices [11, 29–32], quantify investment costs and profit perspectives [33], assess honey properties, quality and commercialization routes [12], rear queens artificially [34], and diagnose the overall situation of the sector in different regions [17, 31, 35]. However, no attempt has yet been made to relate production and income indicators to management practices and other confounding variables across a large geographic scale.

Here we profited from the large diversity of meliponiculture practices found in Brazil [2, 11], to assess the impact of particular management practices on productivity and economic revenues from the commercialization of stingless bee products. Our study represents the first large-scale effort aiming at optimizing meliponiculture using a quantitative approach.

Materials and Methods

Survey data

A draft survey questionnaire was designed in 2012 in collaboration with several researchers and successful beekeepers from Brazil. The survey included questions on the species kept, the number of colonies, management techniques, and sales of bee products, among others, as well as an informed consent to participate in this study that was signed by all participants. As our study did not involve indigenous people, and does not disclose any personal information from its participants, we did not seek approval by an ethics committee. This initial questionnaire was tested across Rio Grande do Norte, a small Brazilian State with a rich tradition in meliponiculture [35]. The analysis of the data retrieved from this initial examination allowed us to refine the survey, eliminating meaningless questions, adding new ones, and rewording to obtain more accurate answers (see S1 Questionnaire for final questionnaire). We used this questionnaire to personally interview several beekeepers from different Brazilian States. A pdf version of the final questionnaire was also distributed to research institutions and extension officers throughout Brazil, whom interviewed local beekeepers. In addition, an online version of the survey (created using Google Forms) was placed in the University of São Paulo´s Bee Lab website, and disseminated through the main Brazilian social networks related to meliponiculture (Yahoo and Facebook groups). The project was presented in local meliponiculture conferences and the survey promoted via an article published in the main beekeeping journal of Brazil [36]. Because many Brazilian stingless beekeepers live in remote rural areas lacking internet, telephone, and access to postal services, we were not able to use these communication channels as effectively as previous surveys undertaken in Australia [17, 37].

Survey data was collected during one year, between January 2013 and January 2014. Only data from beekeepers having at least one stingless bee colony at the moment of the interview were included in this study. Although respondents might have provided false or inaccurate answers in some cases, we have no reason to expect systematic response biases that could compromise our analyses. To minimize such potential biases, we carefully curated the data eliminating duplicated entries, contacting some beekeepers again to confirm certain answers, cross validating replies, and checking for outliers in each response. Four outlier observations were deleted because they were unrealistic, based on the other information provided and our current knowledge in meliponiculture (see S1 Dataset Rscripts for final dataset).

Statistical analyses

A model selection approach was used to relate production and income indicators to management practices and other confounding variables. Such an approach has gained substantial support in the natural sciences during the last decade, and is particularly well suited to analyze complex datasets, when several different competing hypotheses can be put forward [38, 39]. Because we expected production and income to be mainly determined by the ability of beekeepers to multiply colonies, sell colonies, and sell honey, we first constructed three logistic models to identify which predictors explained whether beekeepers multiply colonies, sell honey, or sell colonies. We then constructed nine additional models relating the same predictors to nine different continuous indicators of productivity and income. We considered thirty-nine different predictors (categorical and continuous) which described the beekeepers' social background, property characteristics, beekeeping experience, management practices, and other confounding factors (see S1 Table for details). For some models we also included the total number of colonies kept as an additional predictor, to account for variation in the size of beekeeping operations. In each model, we first constructed model subsets containing groups of

related predictors (education, property characteristics, or management practices), and included the main species as a random effect (assuming different species vary in optimal management practices). We initially considered groups of predictors separately, because the data contained many missing observations for particular combinations of predictors, and thus we could not construct one single full model. For each subset of predictors, a full model containing all or most predictors was compared to reduced models where each predictor was eliminated one by one. A predictor was excluded if the reduced model (lacking that particular predictor) was not significantly different from the full model (using likelihood ratio tests, alpha = 0.05). Predictors were eliminated sequentially, based on the magnitude of *p*-values, and each time a predictor was eliminated we constructed a new full model with the larger dataset (including the observations that were missing in the dropped predictor) and repeated the whole model selection process. All statistical comparisons between two models were conducted using the same dataset. To avoid overfitting the data, care was taken not to construct models containing less than 15 observations per predictor. Once we identified the key predictor variables in each subset, we constructed a complete-cases dataset with these predictors and ran a similar model selection protocol to obtain a final model. Interaction effects were included in these final models to test whether they improved the models without interactions. Finally, we used the Akaike Information Criterion (AIC) to compare the best models with and without random intercepts, and in some cases with a random slope (where we had *a priori* justification for considering a differential response to predictors across bee species). The best models were validated by plotting residual vs. fitted values, residual vs. predictors, by looking at the distribution of residuals, by checking for multicollinearity, and by comparing observed data to simulations from the models. All analyses and graphs were implemented with R, version 3.1.0 [40] (R-scripts for all models are available in S1 Dataset Rscripts).

Results

The final data set consisted of 251 observations from stingless beekeepers distributed across 20 Brazilian States (S2 Table). Men dominated the data set (only 14 of the interviewed beekeepers were woman), and nearly half of all respondents had jobs within the agriculture sector, were public servants or pensioners. The age of beekeepers ranged between 15 and 80, while the time keeping bees varied between less than a year and 54 years (see S3 Table for summary statistics). The total number of colonies kept by beekeepers ranged between 1 and 3500, with the majority keeping less than 100 colonies (Fig 1). While 66% of beekeepers multiplied at least one colony per year, only 25% sold colonies, 30% sold honey, and 14% sold both colonies and honey during the previous year. Although we registered a total of 19 species kept by the surveyed beekeepers, the most commonly kept species were *Tetragonisca angustula*, *Melipona quadrifasciata*, *M. scutellaris*, *M. subnitida, and M. fasciculata* (Fig 2, S4 Table).

Experience, technical skills related to bee management, and some property characteristics were found to be significant predictors of whether beekeepers multiply colonies, sell colonies, or sell honey (Table 1). The best models for nine different continuous indicators of productivity and income are summarized in Table 2. Regression coefficients, *p*-values, and confidence intervals for all models are summarized in S5 Table, while S1 and S2 Figs show the relationships between response and predictor variables respectively. Years spent keeping bees and the number of known beekeepers were the most consistently included predictors in the best models. The number of colonies sold, the liters of honey sold, and the total number of hives kept were all positively associated with years spent keeping bees (Fig 3A). The number of known beekeepers was also positively associated with the total number of colonies kept and the number of multiplied colonies per year (Fig 3B). Beekeepers feeding their colonies with sugar syrup or

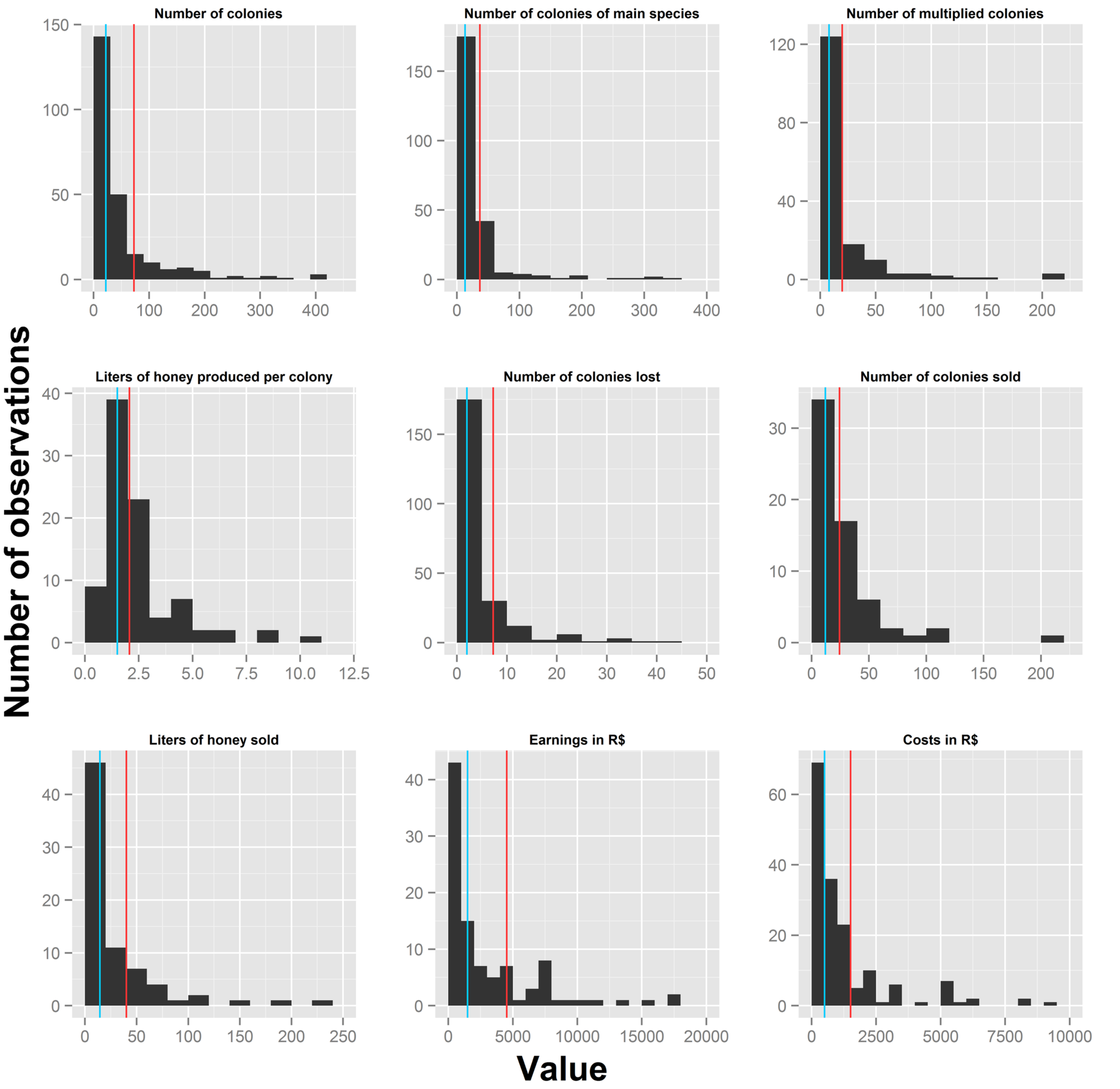

Fig 1. Histograms showing the frequency distribution of nine continuous indicators of productivity and income. Red bars represent the means, while blue bars show the median values. Extreme values of some variables were excluded to improve clarity (see S3 Table for full summary statistics).

doi:10.1371/journal.pone.0121157.g001

honey had a larger number of colonies and multiplied more colonies per year (Fig 4A), whereas beekeepers employing established honey conservation methods sold more honey and had higher yearly earnings than those storing honey in a refrigerator or simply leaving it outside (Fig 4B). Beekeepers who inspected their colonies more often and harvested honey with a

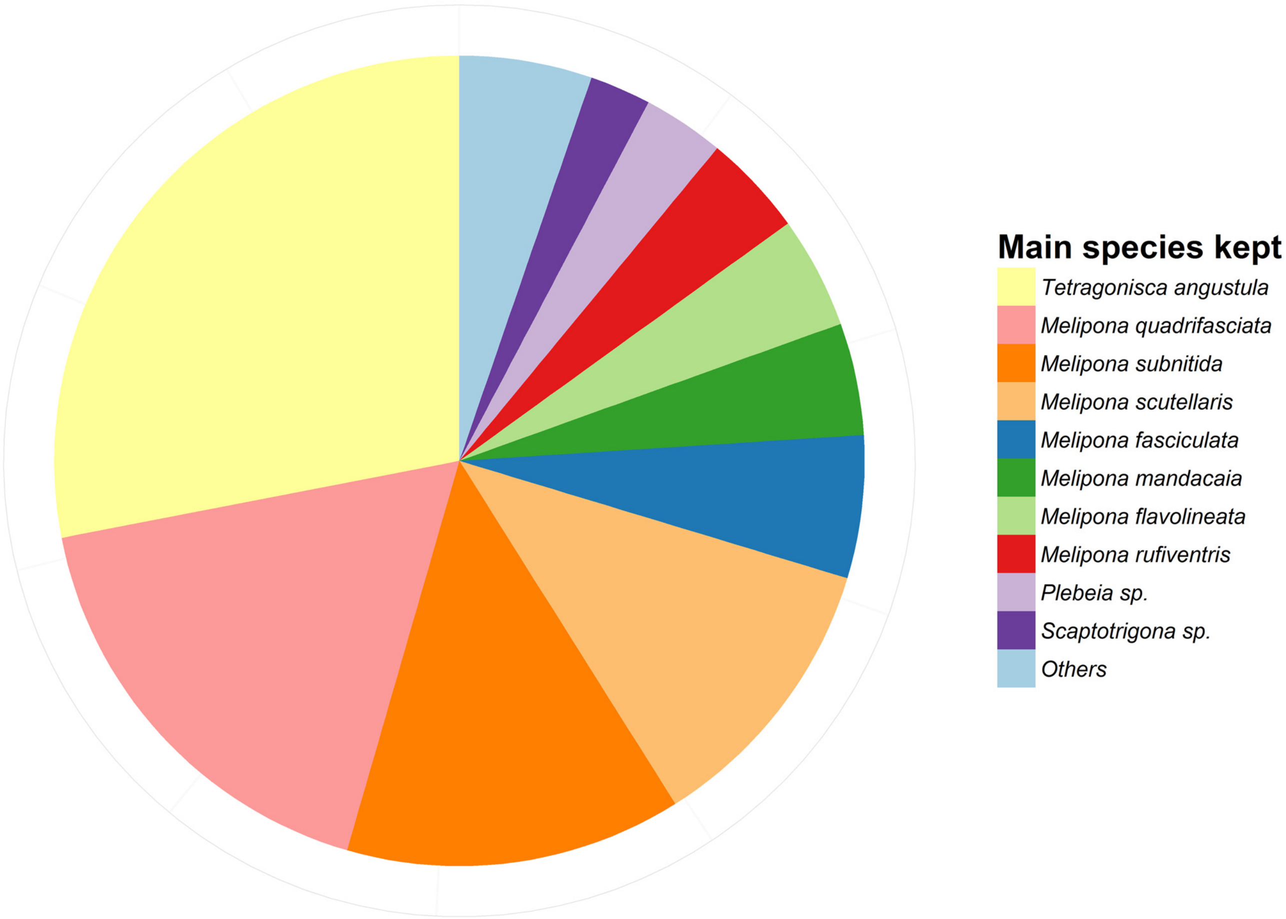

Fig 2. Proportional representation of the main species kept by 246 Brazilian stingless beekeepers (See S4 Table for sample sizes and the complete species list).

doi:10.1371/journal.pone.0121157.g002

Table 1. Best models describing whether beekeepers multiply colonies, sell colonies or sell honey.

Response	N	Model		Interpretation
		Fixed effects	Random effects	
**Multiplies colonies?**	203	Number of colonies, Meliponiculture course, Native vegetation, and Supplementary feeding	Main species	Colony multiplication is more frequent among beekeepers that have more colonies, did a course in meliponiculture, keep their bees within 3 Km of native vegetation, and feed their colonies. These trends hold across the main species kept.
**Sells colonies?**	206	Sells honey?, Number of known beekeepers, and Supplementary feeding	Main species	Selling colonies is more frequent among beekeepers that sell honey, know a larger number of other beekeepers, and feed their colonies. These trends hold across the main species kept.
**Sells honey?**	198	Sells colonies?, Years keeping bees, Meliponiculture course, Education level, Crops, and Property type	Main species	Selling honey is more frequent among beekeepers that sell colonies, have more years of experience keeping bees, did a course in meliponiculture, have a lower level of education, have crops on their property, and have a rural property. These trends hold across the main species kept.

The number of observations included in each model is provided (N) along with the model structure and its biological interpretation. All models are generalized linear mixed models (GLMM) with a Bernoulli distributed response variable (logistic regressions). Regression coefficients, *p*-values, and confidence intervals for all models are summarized in S5 Table.

doi:10.1371/journal.pone.0121157.t001

Table 2. Best models describing nine different indicators of productivity and income (response variables).

Response	N	ModelType[a]	Model		Interpretation
			Fixed effects	Random effects	
**Number of colonies**	187	LMM	Years keeping bees, Number of known beekeepers, Native vegetation, Use of vinegar, and Supplementary feeding	Main species	Beekeepers with more years of experience that know a larger number of other beekeepers, use vinegar to control parasitic flies and feed their colonies, have more colonies. The number of colonies is also higher in locations that have native vegetation within 3 Km. These effects hold across the main species kept.
**Number of colonies of main species**	195	LMM	Years keeping bees, and Number of known beekeepers	Main species	Beekeepers with more years of experience that know a larger number of other beekeepers, have more colonies of the principal species. These effects hold across the main species kept.
**Number of multiplied colonies**	126	LMM	Number of colonies, Number of known beekeepers, Supplementary feeding, and Property ownership	Main species	Beekeepers with more colonies, that know a larger number of other beekeepers, and feed their colonies, manage to multiply a larger number of colonies per year. Property owners multiply fewer colonies per year than not-owners. These effects hold across the main species kept.
**Liters of honey produced per colony**	70	LMM	Selective breeding	Main species	Honey production per colony is higher among beekeepers that multiply their colonies selectively. This effect holds across the main species kept.
**Number of colonies lost**[b]	86	GLMM	Inspection frequency and Honey harvest method	Individual, Main species	Beekeepers that inspect their colonies less frequently and harvest honey by flipping the boxes lose more colonies. These effects hold across the main species kept.
**Number of colonies sold**	60	LMM	Years keeping bees	Main species	Beekeepers with more years of experience keeping bees, sell more colonies per year. This effect holds across the main species kept.
**Liters of honey sold**	64	LMM	Number of colonies of main species, Years keeping bees, and Honey conservation method	Years keeping bees, and Main species	Beekeepers with more colonies of the main species, more years of experience keeping bees, and using an established honey conservation method, sell more honey per year. These effects hold across the main species kept, although the magnitude of the relationship between Years keeping bees and Liters of honey sold varies.
**Earnings in R$** [3]	75	LM	Number of colonies, Labeling of honey containers, Honey conservation method and the interaction Number of colonies-Labeling of honey containers	-	Beekeepers with more colonies that label honey containers and use an established honey conservation method, have higher yearly earnings. The influence of the number of colonies on earnings is more pronounced among beekeepers that label honey containers. These effects do not vary across the main species kept.
**Costs in R$** [c]	132	LM	Number of colonies, Education level, Feeding frequency, Age, and the interaction Feeding frequency-Age	-	Yearly costs increase with the number of colonies, the level of education, the feeding frequency, and the age of beekeepers. Older beekeepers spend more money regardless how frequently they feed their colonies. These effects not vary across the main species kept.

The number of observations included in each model is provided (N) along with the model type, the model structure and its biological interpretation. Regression coefficients, *p*-values, and confidence intervals for all models are summarized in S5 Table.

[a] Linear model (LM), linear mixed model (LMM), or Generalized linear mixed model (GLMM).

[b] Number of lost colonies per year standardized by the total number of colonies kept (used as an offset). GLMM with a Poisson distribution. Overdispersion accounted for by including individual as a random effect.

[c] Main species kept was excluded as a random effect since its variance approached zero in a mixed model.

doi:10.1371/journal.pone.0121157.t002

syringe lost fewer colonies per year (Fig 5A and 5B). The use of vinegar to control parasitic flies was associated with a larger number of colonies (Fig 5C), and selective breeding was found to increase honey production per colony (Fig 5D). The total number of colonies kept was a key predictor of earnings. However, the relationship between earnings and the number of colonies was steeper among beekeepers labeling honey containers than among those that did not employ labels (Fig 6A). Similarly, we found that costs were explained by an interaction between

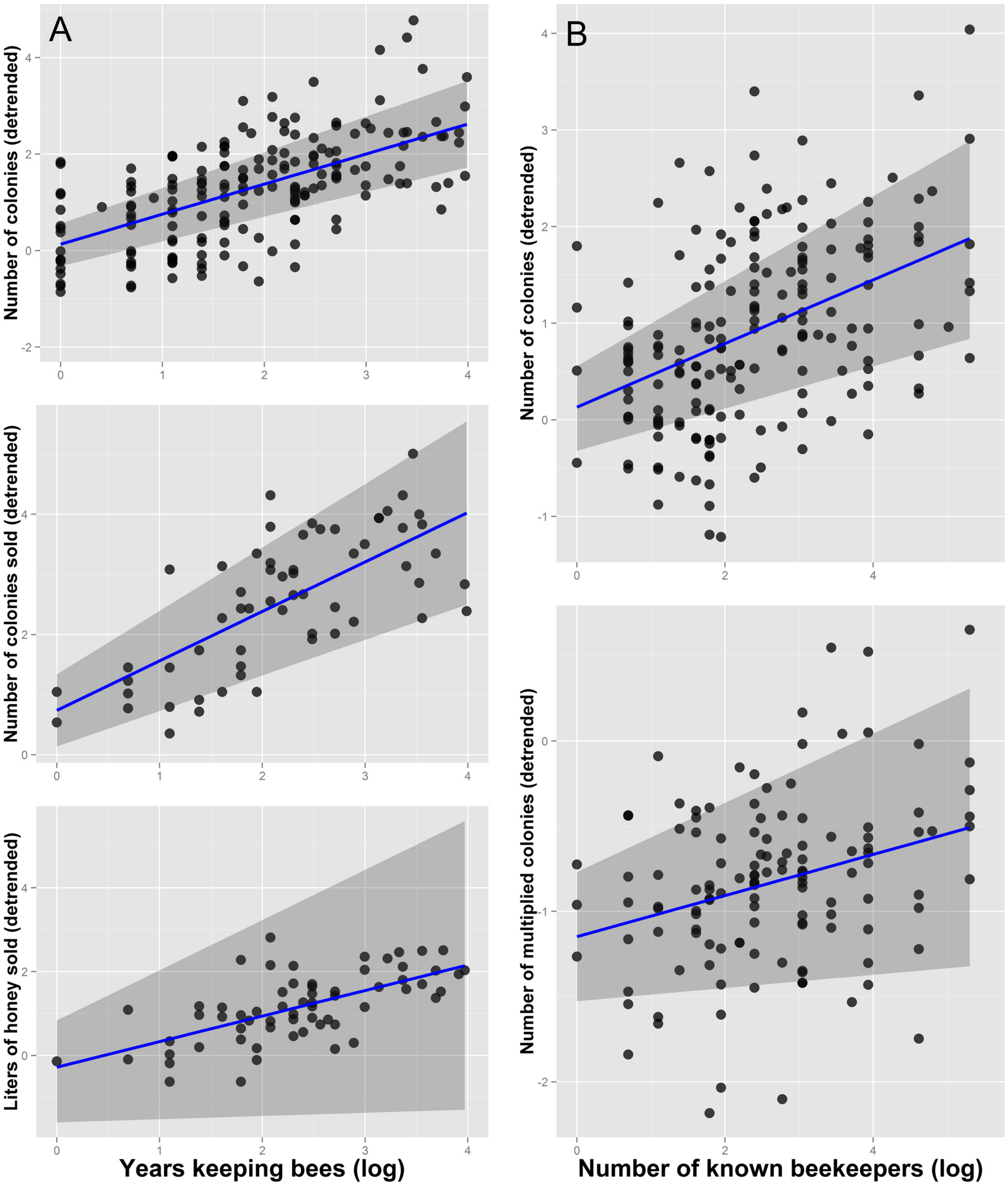
A
B
Number of colonies (detrended)
Number of colonies sold (detrended)
Liters of honey sold (detrended)
Number of multiplied colonies (detrended)
Years keeping bees (log)
Number of known beekeepers (log)

Fig 3. Influence of the years of experience keeping bees on the number of colonies kept, the number of colonies sold and the liters of honey sold (A), and influence of the number of known beekeepers on the number of colonies kept and the number of multiplied colonies (B). Response variables are detrended to show the correct relationship between response and particular predictor variables (the effect of the other predictor variables has been subtracted out). Blue lines represent fitted curves while gray areas show 95% confidence intervals.

doi:10.1371/journal.pone.0121157.g003

the beekeeper's age and feeding frequency, whereby the relationship between age and costs was steeper among beekeepers that did not feed their colonies very often (Fig 6B).

When asked to identify the main problem related to keeping stingless bees, more than half of all interviewed beekeepers pointed at the current legislation and the management skills (Fig 7). Only 13% of the interviewed beekeepers exploited alternative bee products other than honey and colonies, including propolis, pollen, boxes and wax (Fig 8). Finally, when asked to assess the status of wild stingless bee populations, 92% of beekeepers responded they believe there are now fewer wild bees than fifty years ago (this proportion also holds when considering only beekeepers older than 59 years).

Discussion

Survey data from 251 beekeepers scattered across Brazil revealed the influence of specific management practices and other confounding factors over productivity and income indicators. Specifically, beekeeping experience, networking with other beekeepers, and technical skills related to bee management were found to be important predictors of productivity and income, regardless of species-specific differences in management. Even though we focused on the general influence of management practices across species, we would like to stress out the importance of optimizing species-specific management practices. Although not many stingless bee species are actually managed [1], there are more than 400 recognized species in the Neotropics [41], each showing a different life history and distribution range [12, 28]. Further efforts are needed to optimize husbandry of each managed species and facilitate the commercial use of other still unmanaged species. Our results could thus be used as a general guideline to optimize stingless beekeeping, bearing in mind that particular practices might be more or less effective across different species or groups of beekeepers. We also believe that extension programs aiming to optimize stingless beekeeping should consider local or indigenous traditions and beliefs, to enrich these efforts and preserve cultural diversity.

Although we collected data from beekeepers of 20 Brazilian States with varying socio-economic backgrounds, our data set should not be considered a representation of the population of stingless beekeepers in Brazil. There are about 1800 stingless beekeepers registered in the most popular social network (a Yahoo group), but the total number of stingless beekeepers in Brazil is estimated to be much higher (above 5000 according to the administrators of that group), as many live in remote rural areas without internet access and have never been registered by any agency. Nonetheless, we believe our data is suitable to model the relationships between management practices and production/income indicators. For instance, we did not find a strong dependency structure among predictors (S2 Fig.), so our models are appropriate to assess the response of stingless bees to different management practices. Moreover, our study is the most extensive assessment of stingless beekeeping undertaken in a developing country [15, 31, 35, 42], and the first large-scale effort aiming at optimizing stingless beekeeping for honey/colony production based on quantitative data.

Multiplication of colonies

Beekeepers can acquire new colonies either by collecting nests in the wild, by capturing new swarms using trap-nests [43], by buying them from other beekeepers, or by multiplying their

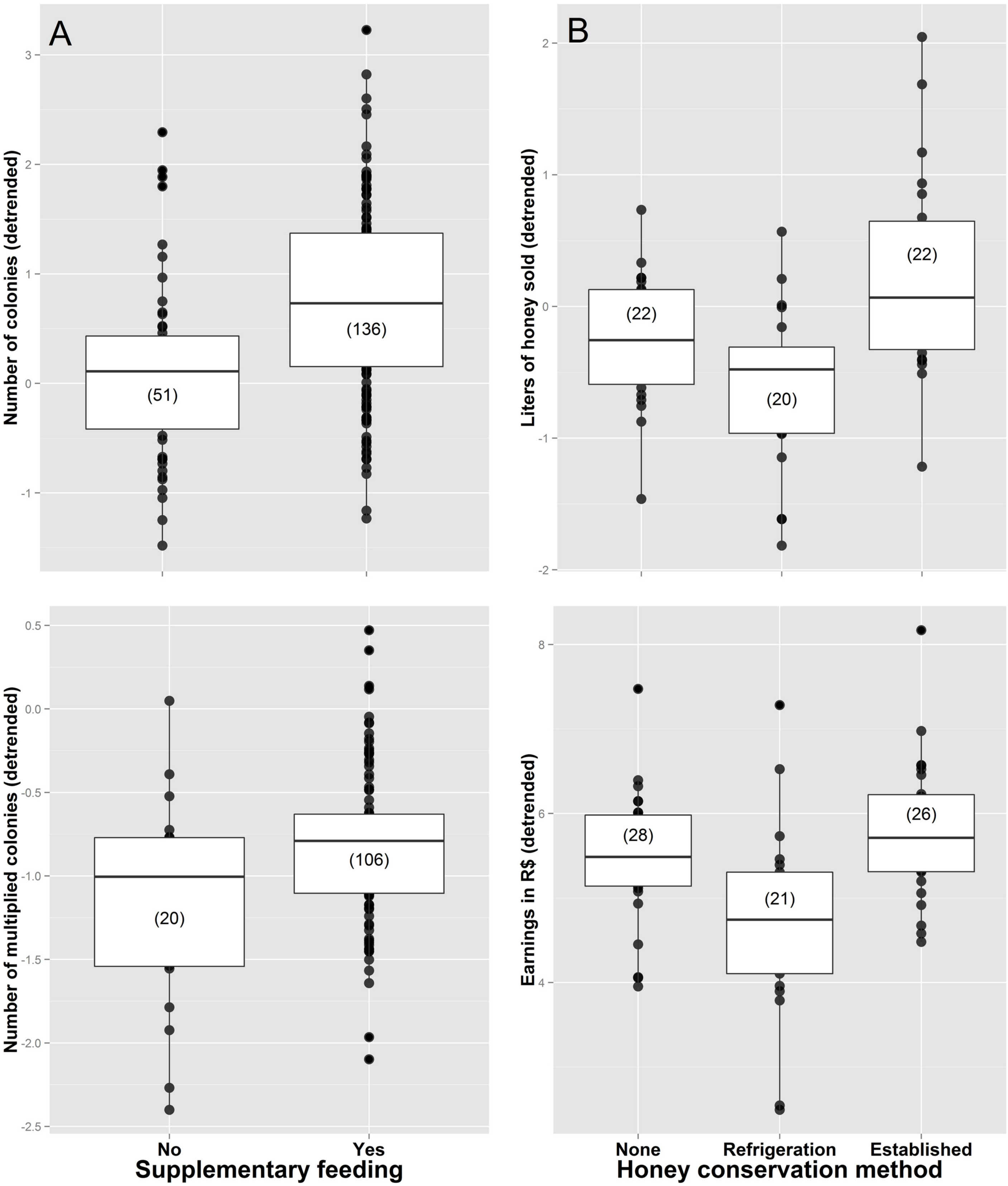
A
B
Number of colonies (detrended)
Liters of honey sold (detrended)
Number of multiplied colonies (detrended)
Earnings in R$ (detrended)
(51)
(136)
(22)
(20)
(22)
(20)
(106)
(28)
(21)
(26)
No
Yes
Supplementary feeding
None
Refrigeration
Established
Honey conservation method

Fig 4. Influence of supplementary feeding on the number of colonies and the number of multiplied colonies (A), and influence of the honey conservation method on the liters of honey sold and yearly earnings (B). Established methods include freezing, pasteurization, maturation and dehumidification. Response variables are detrended to show the correct relationship between response and particular predictor variables (the effect of the other predictor variables has been subtracted out). Median values are represented by the lines inside boxes, which span the first and third quartiles. Dots show all observations outside these quartiles, and sample sizes are provided in brackets.

doi:10.1371/journal.pone.0121157.g004

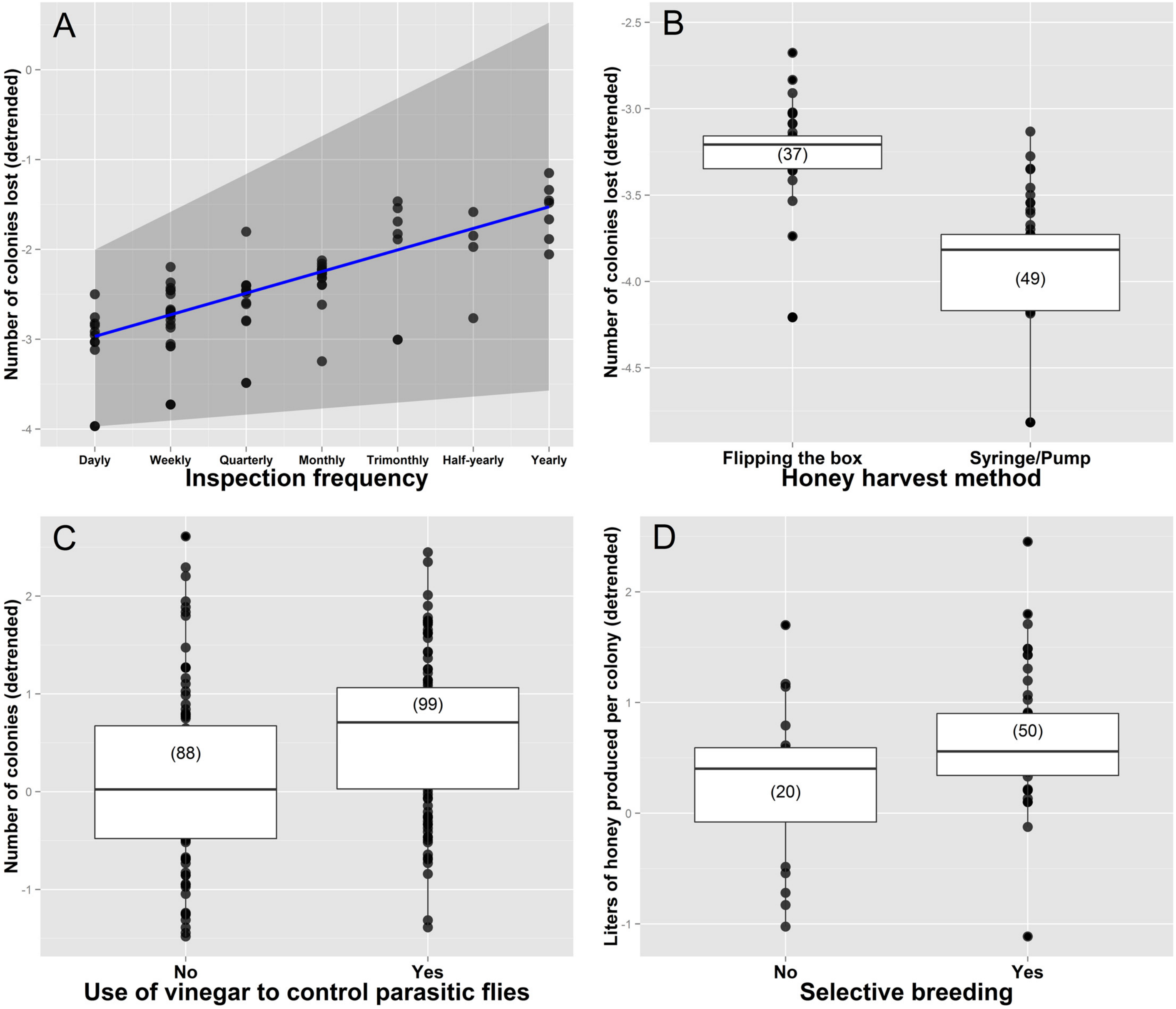

Fig 5. Influence of colony inspection frequency on the number of colonies lost (A), influence of the honey harvest method on the number of colonies lost (B), influence of the use of vinegar on the number of colonies (C), and influence of selective breeding on the liters of honey produced per colony (D). Response variables are detrended to show the correct relationship between response and particular predictor variables (the effect of the other predictor variables has been subtracted out). In A, the blue line represents the fitted curve while the gray area shows the 95% confidence interval. In B, C and D, median values are represented by the lines inside boxes, which span the first and third quartiles. Dots show all observations outside these quartiles, and sample sizes are provided in brackets.

doi:10.1371/journal.pone.0121157.g005

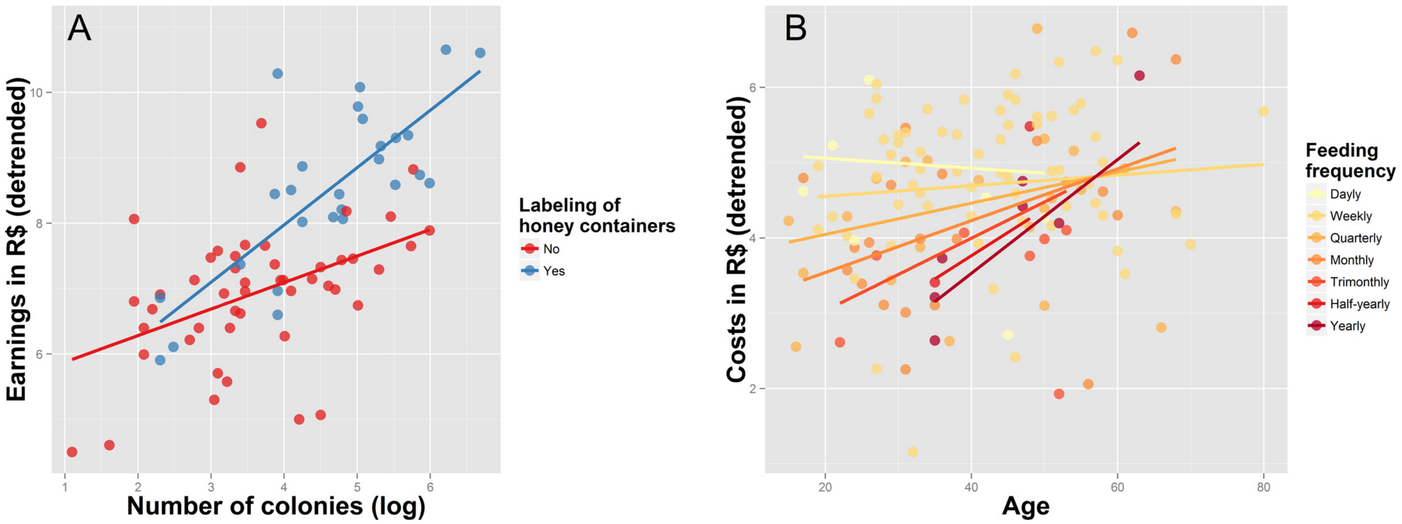

Fig 6. Influence of the number of colonies on yearly earnings, among beekeepers that label and do not label honey containers (A), and influence of the beekeeper's age on yearly costs, among beekeepers that feed their colonies with varying frequencies (B). Response variables are detrended to show the correct relationship between response and particular predictor variables (the effect of the other predictor variables has been subtracted out). Lines represent fitted curves.

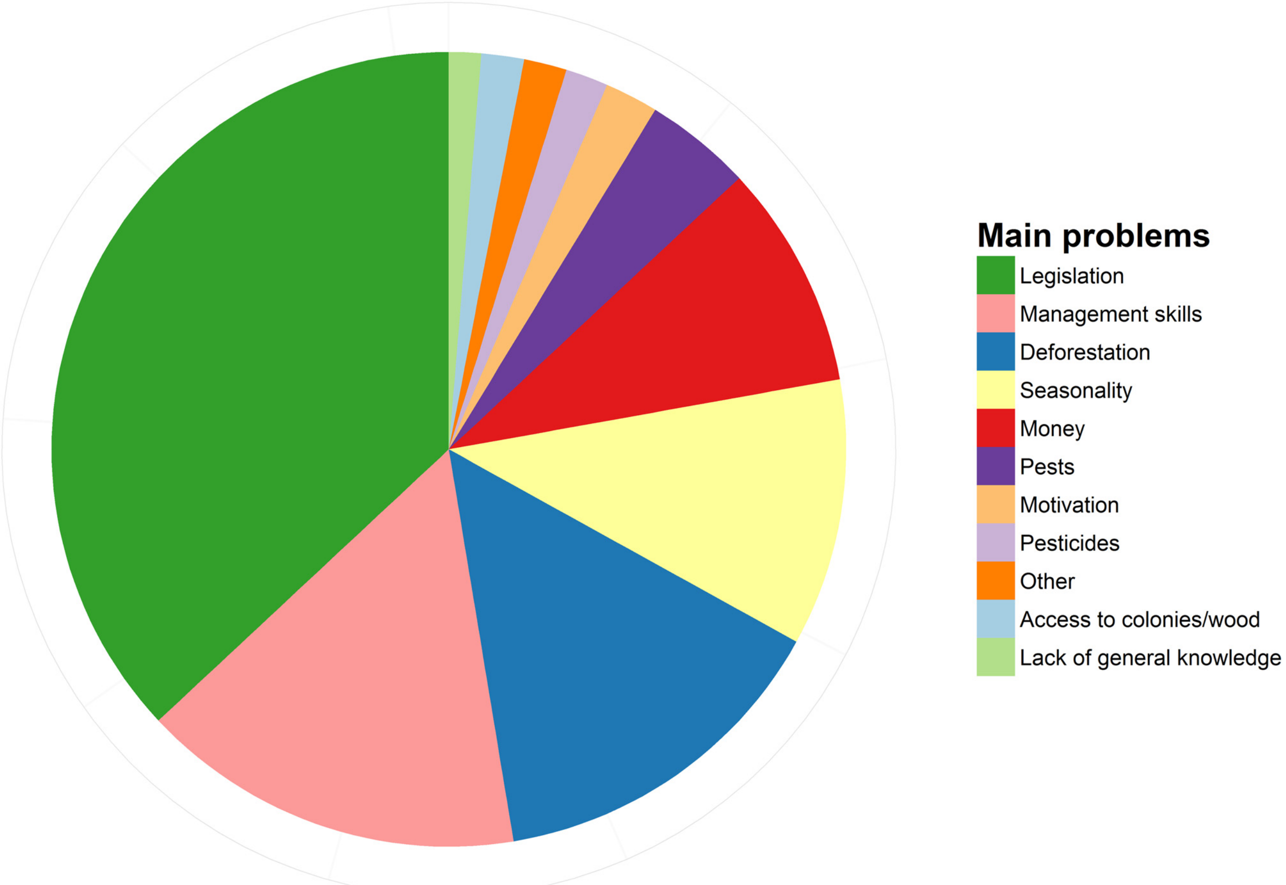

Fig 7. Proportional representation of the main problems affecting stingless beekeeping in Brazil, as identified by 230 beekeepers.

doi:10.1371/journal.pone.0121157.g007

doi:10.1371/journal.pone.0121157.g006

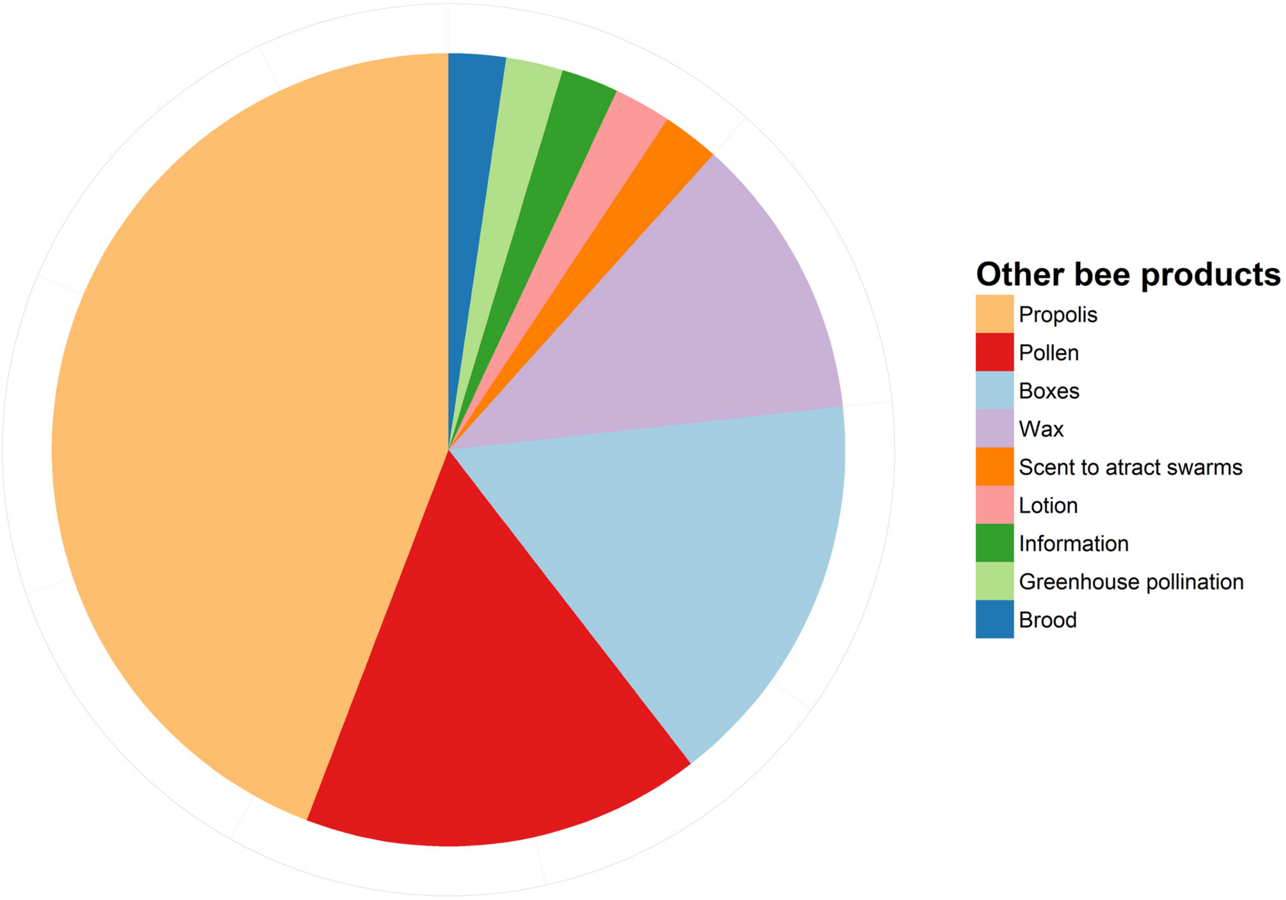

Fig 8. Proportional representation of other bee products commercialized by 13% (32) of all interviewed beekeepers.

doi:10.1371/journal.pone.0121157.g008

own colonies. Colony multiplication, whereby one colony is split into two or more colonies, is considered the most sustainable approach to obtain new colonies, since it involves low economic costs (a new box) and does not require the removal of wild bees from their native habitat [1]. Colony multiplication is a key part of stingless bee management, since more colonies are needed to produce more honey or more colonies to sell. Our results show that beekeepers who received a course in meliponiculture are more likely to multiply colonies than those that did not, highlighting the importance of acquiring the necessary management skills to multiply colonies. This is because multiplying colonies of some species is not easy, requiring knowledge on the best moment to do it, and the ability to recognize and carefully extract the brood discs at the appropriate developmental stage. Moreover, newly multiplied colonies are usually weak and vulnerable to infestation by pests [1]. Our results also indicate that beekeepers keeping their bees close to native vegetation and feeding them with sugar or honey syrup, are also more likely to multiply colonies, which suggests an influence of the available feeding resources on colony multiplication success (see below).

Supplementary feeding and honey conservation method

The importance of supplementary feeding has been highlighted in many technical manuals [11, 29, 44], and it is regarded as an essential part of bee management. Supplementary feeding with sugar syrup or honey has been shown to influence colony growth, performance and

survival, both in stingless bees and in honeybees [45–47]. Such an influence is corroborated by our data, since beekeepers feeding their colonies with sugar syrup had a larger number of colonies and multiplied more colonies per year (Fig 4A). Our data, however, does not allow separating the relative effect of feeding on colony growth, performance, or survival, because the observed pattern is the outcome of the interplay between them. We hypothesize that in places with a marked seasonality, where flower resources are scarce or completely absent during a given time period, supplementary feeding is particularly important to assure colony survival. The same applies to urbanized areas that have lost most native vegetation. For instance, we found that beekeepers who kept their bees close to native vegetation had more colonies, which could imply that the availability of natural resources influences colony growth and multiplication success. Future studies in arid regions of Northeastern Brazil or severely deforested areas, could further test this hypothesis controlling for other confounding factors not accounted for here. Additional research is also needed to assess the influence of different supplementary feeding diets on colony performance.

Honey conservation method was found to be a key predictor of the amount of honey sold and the beekeeper´s yearly earnings (Fig 4B). It is well known that stingless bee honey ferments much faster than honeybee honey due to its higher water content [48]. For this reason, different conservation methods have been developed to increase post-harvest stability and shelf life of stingless bee honey, including freezing, pasteurization, maturation, and dehumidification or dehydration [49]. Our findings highlight the importance of honey conservation, as beekeepers employing any of these established honey conservation methods sold more honey and earned more money than those that did not. Interestingly, our results also show that beekeepers leaving honey in the refrigerator (which is not considered an established conservation method), sold even less honey and earned less money than beekeepers that left honey out of the refrigerator. This finding could be explained by the beekeepers or their families being more tempted to eat the honey when found in the refrigerator, thus reducing the stock and increasing its exposure to potential contaminants.

Inspection frequency, honey harvest method, and the use of vinegar to control parasitic flies

Phorid flies (Diptera, Phoridae) constitute one of the most devastating pests of stingless bee colonies [11, 15, 50]. Attracted by the odors emitted by stored pollen, the flies enter colonies and lay hundreds of eggs. These later become voracious larvae that deplete the colony´s food stores, causing a considerable damage and often the total collapse of the colony. To control phorid fly infestations many beekeepers use vinegar traps [1], which consist of small vinegar containers provided with lids pierced with small holes (allowing flies but not bees to enter). Vinegar attracts the flies, which enter the containers and drown. Our results show that beekeepers employing such traps have a larger number of colonies than those that do not (Fig 5C), suggesting a relationship between the use of vinegar traps and colony growth and survival. Experimental work directly measuring colony growth and survival in colonies with and without vinegar traps is needed to support this pattern.

Although the use of vinegar traps was not found to be among the factors explaining colony losses (Table 2), the predictors explaining colony losses (inspection frequency and honey harvest method) are indirectly related to phorid fly infestation. Beekeepers that check their colonies frequently are able to detect infestations and control them before they become a major problem. In addition, they can also strengthen weak colonies with workers or brood from stronger ones, provide supplementary feeding if needed, or detect when a colony is queen-less. Frequent colony inspections thus seem important to minimize colony losses (Fig 5A). Honey

harvest method was also found to influence colony losses. Beekeepers that collect honey by "flipping the box", not only kill a larger portion of the colony´s worker population, but also make the harvested colonies more attractive to phorid flies. This is because this technique involves the rupture and exposure of honey and pollen pots before flipping the box to let the honey drain into a collection container. The harvested colonies are thus left with exposed pollen, which makes them particularly attractive to phorid flies. This can explain why beekeepers employing a syringe or an electric pump to harvest honey, which cause a minor rupture of honey pots and leave pollen deposits untouched, have much lower colony losses (Fig 5B).

Labeling of honey containers and feeding frequency

Economic gains from selling honey or colonies were determined by the total number of colonies kept. This is not surprising, given that more colonies imply more honey and more colonies are available to sell. However, the practice of labeling honey containers was found to affect this relationship in an unexpected way. For instance, the relationship between earnings and the number of colonies was much stronger among beekeepers labeling honey containers than among those that do not employ labels (Fig 6A). This result highlights the importance of labeling honey containers (or investing more effort in branding the product), as a strategy to increase the yearly earning of beekeepers selling honey.

On the other hand, costs associated to keeping bees were found to be influenced by feeding frequency. Because the acquisition of sugar or honey for supplementary feeding implies input costs, our results could be explained by the fact that beekeepers feeding their bees more frequently need to buy more sugar or honey. Interestingly, we also found that this pattern was affected by the age of beekeepers. In general older beekeepers invested more into their bees, regardless how many colonies they kept and how frequently they feed them. Hence, older beekeepers seem to be more involved with managing their colonies, investing more money into things other than supplementary feeding, such as buying more colonies, building better boxes or improving the condition of their bee business.

Selective breeding

Selective bee breeding has had a tremendous impact on apiculture, resulting in healthier and more productive bees [51, 52]. In stingless bees, no formal breeding programs have been implemented yet, and there is surprisingly little data on queen rearing and other breeding techniques [34]. Here we show that beekeepers undertaking some selective breeding have colonies that produce more honey (Fig 5D). It should be pointed out, however, that we did not assess whether beekeepers preferentially multiplied a selection of their colonies based on desirable traits (such as honey production, colony health, worker population, etc.). We only recorded if beekeepers were able to keep track of the genetic origin of their colonies by avoiding to mix brood from different colonies during colony multiplications. Mixing brood is a common practice, since the easiest way to strengthen a weak colony is offering it fresh brood from another (usually stronger) colony. Such mixing can result in the replacement of the original queen by a new queen, born from the newly introduced brood disc, thus making it impossible for the beekeeper to track maternal lineages. Our results should thus be interpreted as an association between the ability to maintain and track such maternal lineages and colony honey production [but see [53]].

Beekeeping experience and network of known beekeepers

Only a minority of the surveyed beekeepers sold honey (30%) or colonies (25%), a fact that illustrates that stingless beekeeping is still mainly a non-commercial practice. Motivating

beekeepers to sell their products is essential to transform meliponiculture into a profitable activity. We found that the years of beekeeping experience and the number of known beekeepers are key determinants of whether beekeepers sell colonies or honey (Table 1). Years keeping bees was found to be an important predictor of the total number of hives kept, as well as the number of colonies sold and the liters of honey sold (Fig 3A). Beekeepers with more experience are likely to have acquired a broader set of technical skills throughout their lives. For instance, many beekeepers make and test different boxes, and try different sugar syrup recipes, feeding techniques, pest control methods, and many other management practices. More experienced beekeepers thus have had more time to test different methods, and establish the most effective set of management practices [15]. Likewise, more experience beekeepers have had more time to build a larger network of known beekeepers (S2 Fig.). Such network is important, as we show that the number of known beekeepers was associated to the total number of colonies kept and to the number of multiplied colonies per year (Fig 3B). The network of known beekeepers thus seems to be a source of technical knowledge on how to multiply colonies, as well as a source of colonies. Indeed, many beekeepers exchange knowledge and colonies, and some leading beekeepers are well known as promoters of meliponiculture, offering free hives and training to starters and amateurs across vast regions [11, 54].

Interestingly, years of experience but not the network of known beekeepers was found to predict the number of colonies sold, the liters of honey sold, and whether beekeepers sold honey or not, suggesting that technical skills are the limiting factor determining sales. For instance, beekeepers that participated in a meliponiculture course were more likely to sell honey (in any amount). This explanation is further supported by the fact that demand for stingless bee products often exceeds supply [3, 16], so sales seem to be limited by the beekeeper´s ability to produce enough honey or colonies. However, beekeepers cultivating crops on their property were found to be more likely to sell honey, indicating a possible role of the exposure to other agro-markets. The lack of an association between the number of known beekeepers and the probability of selling honey, can also be explained by the fact that contacting other beekeepers is more difficult in rural areas than in urban centers (honey sales were indeed found to be more common in rural areas). On the other hand, beekeepers with a larger network of known beekeepers were found to be more likely to sell colonies, a result that suggests that beekeepers are trading colonies among themselves. Indeed, many beekeepers confirmed that they had sold their colonies to other fellow beekeepers (S3 Table). Because the current legislation restricts the commercialization of colonies (see below), beekeepers might be more tempted to sell colonies to trusted beekeepers.

Main problems and opportunities

When asked to identify the main problem related to keeping stingless bees, more than half of all interviewees pointed at the current legislation and the lack of management skills (Fig 7). This is not surprising since the current Brazilian legislation severely restricts the commercial use of native bees and their products, imposing compulsory registration for stingless beekeepers with more than 50 colonies or those who wish to commercialize any bee products, and outlawing the unauthorized transportation of colonies [55]. Registrations and applications for transport permits are cumbersome and require beekeepers from remote rural areas to travel to urban centers. Currently, there is a movement to change this legislation, and some states have taken the lead in implementing regional regulations [56]. However, a change at the federal level is urgently needed to ease these restrictions, and acknowledge stingless beekeeping as a conservation and development tool. On the other hand, the need for more efficient management skills calls for more research devoted to optimize management practices, as well as more

extension work to make the knowledge resulting from such research available to beekeepers [1, 31].

We foresee an enormous potential in the stingless beekeeping industry. Once management practices are optimized and the activity is decriminalized, we expect that the market for stingless bee products will experience a significant expansion. Although stingless bee honey is frequently commercialized as medicine in developing countries like Brazil, Mexico and Bolivia, there seems to be a growing number of consumers that regard the product as a luxury item and pay much more for it than for conventional honeybee honey [16]. Moreover, the market for organic or special honey, pollen, wax and propolis is growing across Europe and the US [57]. Likewise, there seems to be a high demand for stingless bee colonies to supply other beekeepers, hobbyists and research/education institutions [17, 58]. Although some of the interviewed beekeepers (13%) already exploit alternative bee products such as propolis, pollen, boxes, and wax (Fig 8), only one beekeeper offered greenhouse pollination services. In contrast, many stingless beekeepers in Australia already provide commercial pollination services [17]. We expect the number of stingless beekeepers offering pollination services to grow in the near future, as demand for pollination services increases [21].

The assessment of the status of wild stingless bee populations made by the interviewed beekeepers (92% believe there are now less wild bees than 50 years ago), supports recent reports on global bee declines [18, 59, 60]. We must point out that this was no quantitative assessment, as it was based entirely on the beekeeper´s opinion. However, we believe this opinion is informative, given that most beekeepers have a close relationship with their bees, constantly asses the natural resources they use, and frequently collect wild colonies. Many beekeepers map the location of wild colonies, and some of them keep track of a remarkable number of them. Our findings thus imply that many wild stingless bee populations might have declined during the last decades, although the factors driving these declines remain unknown. Like honeybee beekeepers, who have helped maintain honeybee populations in places were wild honeybees have all but disappeared [19], stingless beekeepers could help maintain or increase stingless bee populations in many tropical regions. Incentivizing colony multiplication by stingless beekeepers could be a strategy to increase bee populations and minimize the need to extract colonies from the wild.

Conclusions

Our findings have important implications for the stingless beekeeping industry. We identified particular management practices, which could help many beekeepers produce and sell more honey and colonies and earn more money, thus making the activity more profitable. Specifically, our results highlight the importance of teaching beekeepers to inspect and feed their colonies, how to best multiply them and keep track of genetic lineages, how to best harvest and preserve the honey, how to use vinegar traps to control phorid fly infestations, and how to add value by branding their honey. Future experimental work could further test the patterns documented here. Our work also stresses out the importance of beekeeping experience, suggesting that future extension programs could profit from engaging more experienced beekeepers in the design and implementation of technical courses. Importantly, our results emphasize the key role of the network of known beekeepers as a source of colonies and technical knowledge. We believe that the creation or consolidation of local and state associations of stingless beekeepers could enhance such networks, facilitating communication between beekeepers, and unifying efforts to push forward the commercialization of stingless bee products. Our work underlines the need for more research devoted to optimize management practices, as well as more extension work to transfer the generated knowledge to beekeepers. Such efforts could help transform

stingless beekeeping into a powerful tool to achieve sustainable development, helping low-income communities improve their living conditions, contributing to the conservation of wild bees and plants, and assuring crop pollination services.

Supporting Information

S1 Dataset Rscripts. Full dataset and R-scripts for all models.
(RAR)

S1 Fig. Correlogram showing the correlation coefficients (lower diagonal) and shape of the relationship (upper diagonal) between the continuous response variables (all log-transformed).
(TIF)

S2 Fig. Correlogram showing the correlation coefficients (lower diagonal) and shape of the relationship (upper diagonal) between the continuous predictor variables (all log-transformed except for Inspection and Feeding frequency).
(TIF)

S1 Questionnaire. Questionnaire used to interview beekeepers.
(PDF)

S1 Table. Description of all response and predictor variables.
(PDF)

S2 Table. Gender, State, and Job distribution of interviewed beekeepers.
(PDF)

S3 Table. Summary statistics for all variables. N_{tot} represents the total number of beekeepers providing an answer for each question (not all beekeepers responded to all questions), while N_{reply} represents the number of beekeepers in each reply category (only for categorical variables).
(PDF)

S4 Table. List of the main species kept by 246 Brazilian stingless beekeepers, their common names in Brazil, and the number and proportion of beekeepers rearing each one. Although beekeepers also reared other species, they were not included in this list since they were not registered as the main species kept.
(PDF)

S5 Table. Regression estimates, standard errors (SE), confidence intervals (CI), *z* or *t*-values, and *p*-values for all best models. Estimates are provided for each level of the categorical predictors. Note that all predictors included in these models significantly increased the model ´s likelihood at $p < 0.05$ (likelihood ratio tests).
(PDF)

Acknowledgments

We thank all the beekeepers that participated in this study. Cândida Lima, Elias de Marco, Elisângela de Sousa Rêgo, Giuseppe Puorto, João Maria Alves de Paula, Pedro Alfonso Leite, and Samuel Perichon assisted with the collection of part of the data, while Nicola Bradbear, Tereza Giannini, Isis Mejías, Klaus Jaffé, Sheina Koffler, and two anonymous referees helped improve earlier versions of this manuscript.

Author Contributions

Conceived and designed the experiments: RJ. Analyzed the data: RJ NP. Contributed reagents/materials/analysis tools: VIF. Wrote the paper: RJ NP ATC. Organized local surveys: RJ ATC UMM BB CLC GCZ BMF CM MR GV VIF.

References

1. Cortopassi-Laurino M, Imperatriz-Fonseca VL, Roubik DW, Dollin A, Heard T, Ingrid Aguilar I, et al. Global meliponiculture: challenges and opportunities. Apidologie. 2006; 37(2):275–92.
2. Venturieri GC, Alves DA, Villas-Boas JK, Carvalho CAL, Menezes C, Vollet-Neto A, et al. Meliponicultura no Brasil: situação atual e perspectivas futuras. In: Imperatriz-Fonseca VL, Canhos DAL, Alves DA, Saraiva AM, editors. Polinizadores no Brasil contribuição e perspectivas para a biodiversidade, uso sustentável, conservação e serviços ambientais. São Paulo: EDUSP; 2012.
3. Quezada-Euán J, May-Itza W, González-Acereto J. Meliponiculture in México: problems and perspective for development. Bee World. 2001; 82(4):160–7.
4. Gupta RK, Reybroeck W, van Veen JW, Gupta A. Beekeeping for Poverty Alleviation and Livelihood Security: Springer; 2014.
5. Verma L. Beekeeping in integrated mountain development: economic and scientific perspectives South Asia Books; 1990.
6. Slaa EJ, Sánchez-Chaves LA, Malagodi-Braga KS, Hofstede FE. Stingless bees in applied pollination: practice and perspectives. Apidologie. 2006; 37(2):293–315.
7. Allsopp MH, de Lange WJ, Veldtman R. Valuing Insect Pollination Services with Cost of Replacement. PLoS One. 2008; 3(9):e3128. Artn E3128. doi: 10.1371/Journal.Pone.0003128 PubMed PMID: ISI:000264425700001. PMID: 18781196
8. Kearns CA, Inouye DW, Waser NM. Endangered mutualisms: The conservation of plant-pollinator interactions. Annu Rev Ecol Syst. 1998; 29(1):83–112. doi: 10.1146/annurev.ecolsys.29.1.83
9. Giannini T, Boff S, Cordeiro G, Cartolano Jr E, Veiga A, Imperatriz-Fonseca V, et al. Crop pollinators in Brazil: a review of reported interactions. Apidologie. 2014;online first:1–15. doi: 10.1007/s13592-014-0316-z
10. Biesmeijer JC, Klaus Hartfelder K, Imperatriz-Fonseca VL. Stingless bees: biology and management. Apidologie. 2006; 37(2):121–3.
11. Nogueira-Neto P. Vida e Criação de Abelhas Indígenas Sem Ferrão. São Paulo: Editora Nogueirapis; 1997. 445 p.
12. Vit P, Pedro SR, Roubik D. Pot-honey: a legacy of stingless bees: Springer; 2013.
13. Crane E. Bees and beekeeping: science, practice and world resources: Heinemann Newnes; 1990.
14. Jalil AH. Beescape for Meliponines. Partridge Singapore; 2014
15. Villanueva-Gutiérrez R, Roubik DW, Colli-Ucán W, Güemez-Ricalde FJ, Buchmann SL. A Critical View of Colony Losses in Managed Mayan Honey-Making Bees (Apidae: Meliponini) in the Heart of Zona Maya. J Kans Entomol Soc. 2013; 86(4):352–62.
16. Alves RM. Production and Marketing of Pot-Honey. In: Vit P, Pedro SRM, Roubik D, editors. Pot-Honey: Springer New York; 2013. p. 541–56.
17. Halcroft M, Spooner-Hart R, Haigh A, Heard T, Dollin A. The Australian stingless bee industry: a follow-up survey, one decade on. J Apic Res. 2013; 52(2):1–7.
18. Brown MJF, Paxton RJ. The conservation of bees: a global perspective. Apidologie. 2009; 40(3):410–6. doi: 10.1051/apido/2009019 PubMed PMID: ISI:000267832600014. PMID: 19121515
19. Jaffé R, Dietemann V, Allsopp MH, Costa C, Crewe RM, Dall'olio R, et al. Estimating the density of honeybee colonies across their natural range to fill the gap in pollinator decline censuses. Conserv Biol. 2010; 24(2):583–93. Epub 2009/09/25. doi: 10.1111/j.1523-1739.2009.01331.x PubMed PMID: 19775273.
20. vanEngelsdorp D, Meixner MD. A historical review of managed honey bee populations in Europe and the United States and the factors that may affect them. J Invertebr Pathol. 2010; 103(Supplement 1): S80–S95. doi: 10.1016/j.jip.2009.06.011 PMID: 19909973
21. Aizen MA, Harder LD. The Global Stock of Domesticated Honey Bees Is Growing Slower Than Agricultural Demand for Pollination. Curr Biol. 2009; 19(11):915–8. doi: 10.1016/j.cub.2009.03.071 PubMed PMID: ISI:000266891900024. PMID: 19427214

22. Garibaldi LA, Steffan-Dewenter I, Winfree R, Aizen MA, Bommarco R, Cunningham SA, et al. Wild Pollinators Enhance Fruit Set of Crops Regardless of Honey Bee Abundance. Science. 2013. doi: 10.1126/science.1230200
23. Michener CD. The social behavior of the bees: a comparative study: Belknap Press of Harvard University Press, Cambridge, USA; 1974.
24. Silveira FA, Melo GAR, Almeida EAB. Abelhas brasileiras: sistemática e identificação: Fernando A. Silveira; 2002.
25. Roubik DW. Ecology and natural history of tropical bees: Cambridge University Press; 1992.
26. Buchmann SL, Nabhan GP. The forgotten pollinators: Island Press; 1997.
27. Heard TA. The role of stingless bees in crop pollination. Annu Rev Entomol. 1999; 44(1):183–206.
28. Roubik D. Stingless bee nesting biology. Apidologie. 2006; 37:124–43.
29. Villas-Bôas JK. Manual Tecnológico Mel de Abelhas sem Ferrão. Brasilia: Instituto Sociedade, População e Natureza (ISPN); 2012.
30. Frazão RF. Abelhas Nativas da Amazônia e Populações Tradicionais: Manual de Meliponicultura. Belém, Pará: Instituto Peabiru; 2013.
31. González-Acereto J, Quezada-Euán J, Medina-Medina L. New perspectives for stingless beekeeping in the Yucatan: results of an integral program to rescue and promote the activity. J Apic Res. 2006; 45 (4):234–9.
32. Venturieri GC. Criação de Abelhas Indígenas Sem Ferrão. 2nd ed. Belém: Embrapa Amazônia Oriental; 2008. p. 60.
33. Lobato T, Venturieri GC. Aspectos econômicos da criação de abelhas indígenas sem ferrão (Apidae: Meliponini) no nordeste paraense. In: Oriental EA, editor. Belém: EMBRAPA Amazônia Oriental; 2010.
34. Menezes C, Vollet-Neto A, Fonseca V. An advance in the in vitro rearing of stingless bee queens. Apidologie. 2013; 44(5):491–500. doi: 10.1007/s13592-013-0197-6 PubMed PMID: WOS:000323246700001.
35. Pereira DS, Menezes PR, Belchior V Filho, de Sousa AH, Maracajá PB. Abelhas indígenas criadas no Rio Grande do Norte. Acta Veterinaria Brasilica. 2011; 5(1):81–91.
36. Jaffé R, Madureira-Maia U, Torres Carvalho A, Imperatriz Fonseca VL. Diagnóstico da Meliponicultura no Brasil. Mensagem Doce. 2013; 120:7–9.
37. Heard T, Dollin AE. Stingless bee keeping in Australia: snapshot of an infant industry. Bee World. 2000; 81(3):116–25.
38. Burnham KP, Anderson DR. Model selection and multi-model inference: a practical information-theoretic approach: Springer; 2002.
39. Johnson JB, Omland KS. Model selection in ecology and evolution. Trends Ecol Evol. 2004; 19 (2):101–8. http://dx.doi.org/10.1016/j.tree.2003.10.013. PMID: 16701236
40. R Core T. R: A language and environment for statistical computing. Vienna, Austria: R Foundation for Statistical Computing; 2014.
41. Camargo MF, Pedro SRM. Meliponini Lepeletier, 1836. 2012. In: Catalogue of Bees (Hymenoptera, Apoidea) in the Neotropical Region—online version [Internet]. Available: http://www.moure.cria.org.br/catalogue.
42. Alves R, Justina G, Souza BDA, Dias C, Sodré GDS. Criação de abelhas nativas sem ferrão (Hymenoptera: Apidae): Autosustentabilidade na comunidade de Jóia do Rio, município de Camaçari, estado da Bahia. Magistra; 2006.
43. Oliveira RC, Menezes C, Soares AEE, Fonseca VLI. Trap-nests for stingless bees (Hymenoptera, Meliponini). Apidologie. 2013; 44(1):29–37.
44. Carvalho-Zilse G, Vilas Boas H, Costa K, Nunes-Silva C, Souza M, Fernandes R. Meliponicultura na Amazônia: FINEP/INPA; 2012. 52 p.
45. Dias VHP, Filgueira MA, Oliveira FLd, Dias AM, Costa EMD. Alimentação artificial à base de mel e suas implicações no desenvolvimento de famílias de abelhas jandaíras (Melipona subnitida Ducke) em Mossoró–RN. Revista Verde de Agroecologia e Desenvolvimento Sustentável. 2008;3(3:).
46. Brodschneider R, Crailsheim K. Nutrition and health in honey bees. Apidologie. 2010; 41(3):278–94.
47. Costa L, Venturieri GC. Diet impacts on Melipona flavolineata workers (Apidae, Meliponini). J Apic Res. 2009; 48(1):38–45. doi: 10.3896/ibra.1.48.1.09 PubMed PMID: WOS:000264029800009.
48. Souza B, Roubik D, Barth O, Heard T, Enriquez E, Carvalho C, et al. Composition of stingless bee honey: Setting quality standards. Interciencia. 2006; 31(12):867.

49. Menezes C, Vollet-Neto A, Contrera FAFL, Venturieri GC, Imperatriz-Fonseca VL. The role of useful microorganisms to stingless bees and stingless beekeeping. Pot-honey: a legacy of stingless bees: Springer; 2013. p. 153–71.

50. Roubik DW. Stingless bee nesting biology. Apidologie. 2006; 37(2):124–43.

51. Borst PL. The History of Bee Breeding. Am Bee J. 2012; 152(7):679–83. PubMed PMID: WOS:000305545900012.

52. Bienefeld K, Ehrhardt K, Reinhardt F. Bee breeding around the world—Noticeable success in honey bee selection after the introduction of genetic evaluation using BLUP. Am Bee J. 2008; 148(8):739–42. PubMed PMID: ISI:000257809100020.

53. Wenseleers T, Alves DA, Francoy TM, Billen J, Imperatriz-Fonseca VL. Intraspecific queen parasitism in a highly eusocial bee. Biol Lett. 2011; 7(2):173–6. doi: 10.1098/rsbl.2010.0819 PMID: 20961883

54. Bruening H. Abelha Jandaíra. 3 ed. Natal, RN: SEBRAE/RN; 2006. 138 p.

55. CONAMA. Resolução CONAMA n° 346, de 16 de agosto de 2004. Diário Oficial da União. 2004; 158 (1):70.

56. Carvalho CA, Alves R, Almeida Souza B, Véras S, Alves E, Sodré G. Proposta de regulamento técnico de qualidade fisico-química do mel floral processado produzido por abelhas do gênero Melipona. In: Vit P, Roubik D, editors. Stingless bees process honey and pollen in cerumen pots. Mérida, Venezuela: Facultad de Farmacia y Bioanálisis, Universidad de Los Andes; 2013. p. 1–9.

57. IBCE. Perfil de mercado: Miel de abejas nativas. 2010.

58. Ferreira EA, Paixão MVS, Koshiyama AS, Lorenzon MCA. Meliponicultura como ferramenta de aprendizado em educação ambiental. Ensino, Saúde e Ambiente. 2013; 6(3). doi: 10.1002/jeab.93 PMID: 25004418

59. Potts SG, Biesmeijer JC, Kremen C, Neumann P, Schweiger O, Kunin WE. Global pollinator declines: trends, impacts and drivers. Trends Ecol Evol. 2010; 25(6):345–53. doi: 10.1016/j.tree.2010.01.007 PMID: 20188434

60. González-Varo JP, Biesmeijer JC, Bommarco R, Potts SG, Schweiger O, Smith HG, et al. Combined effects of global change pressures on animal-mediated pollination. Trends Ecol Evol. 2013; 28(9):524–30. http://dx.doi.org/10.1016/j.tree.2013.05.008. doi: 10.1016/j.tree.2013.05.008 PMID: 23746938
